# Inflammatory mediators and lung abnormalities in HIV: A systematic review

**DOI:** 10.1371/journal.pone.0226347

**Published:** 2019-12-12

**Authors:** Breanne M. Head, Ruochen Mao, Yoav Keynan, Zulma Vanessa Rueda

**Affiliations:** 1 Department of Medical Microbiology and Infectious Diseases, University of Manitoba, Winnipeg, Manitoba, Canada; 2 Facultad de Medicina, Universidad Pontificia Bolivariana, Medellín, Antioquia, Colombia; Rutgers University, UNITED STATES

## Abstract

HIV and pneumonia infections have both been shown to negatively impact lung function. However, evidence of the role of inflammation on lung dysfunction in HIV and pneumonia co-infected individuals remains limited. We aimed to systematically review the association of inflammatory markers and lung abnormalities in HIV and pneumonia co-infected individuals. This systematic review was registered with the International Prospective Register of Systematic Reviews on August 15, 2017 (registration number CRD42017069254) and used 4 databases (Cochrane Central Register of Controlled Trials, PubMed Central, Clinical Trials.gov and Google Scholar). All clinical trial, observational, and comparative studies targeting adult (> 18 years old) populations with HIV, pneumonia, or both, that report on immune response (cytokine, chemokine, or biomarker), and lung abnormality as an outcome were eligible. Data selection, risk of bias and extraction were performed independently by 2 blinded reviewers. Due to heterogeneity among the articles, a qualitative synthesis was performed. Our search strategy identified 4454 articles of which, 7 met our inclusion criteria. All of the studies investigated the ability of circulating biomarkers to predict lung damage in HIV. None of the articles included patients with both HIV and pneumonia, nor pneumonia alone. Markers of inflammation (IL-6, TNF-α, CRP), innate defense (cathelicidin), monocyte and macrophage activation (sCD14, sCD163 and, IL-2sRα), endothelial dysfunction (ET-1) and general immune health (CD4/CD8 ratio) were associated with lung abnormalities in HIV. This review highlights the lack of available information regarding the impact of inflammatory mediators on lung function in HIV and pneumonia populations, therefore opportunities to prevent lung damage with available anti-inflammatory treatment or to investigate new ones still remain.

## Introduction

Since the introduction of combination antiretroviral therapy (cART), HIV infection has transitioned from a fatal infection to a chronic and manageable disease, with patient life expectancy approaching that of the general population [1]. However, despite treatment, individuals with HIV continue to have higher rates of morbidity and mortality compared to the general population.

The lungs are a common site of complication during HIV infection, with pneumonia as a leading cause of hospitalization [2–4]. Following HIV establishment within the lungs, there is an increase in monocyte and macrophage activation, inflammatory markers (interleukin [IL]-1, IL-6, IL-8, IL-15, tumor necrosis factor [TNF]-α, granulocyte-macrophage colony-stimulating factor [GM-CSF], and macrophage inflammatory protein [MIP]-lα) [5], and, in contrast to the gut, an increase in CD4+ T cell concentrations [4,6–9]. An efflux of Interferon (IFN)-γ also occurs and functions as a leukocyte chemoattractant, affecting cell differentiation, B cell regulation and natural killer activity [10]. Together, these processes lead to continuous local inflammation and activation, endothelial dysfunction, altered coagulation, and cell destruction [4,6–9,11]. Persistent immune activation can also lead to impaired immunity and lung function decline.

Due to viral replication, a chronic inflammatory state, and impaired immunity, individuals with HIV have significantly greater lung disease compared to healthy individuals [12–17]. In a recent meta-analysis, HIV-infected individuals had a higher prevalence of chronic obstructive pulmonary disease (COPD) compared to HIV-negative individuals (odds ratio, OR 2.58, 95% confidence interval, CI 1.05, 6.35), even after adjusting for tobacco smoking [18]. In addition, Gingo and colleagues [13] found that a forced expiratory volume in 1 second/forced vital capacity (FEV_1_/FVC) ratio <0.7 and a lung diffusing capacity (DLCO) <60% associated with an increase in all-cause mortality in HIV-infected individuals. Decreased diffusing capacity has also been reported in other studies as well [19].

Similarly, in pneumonia, individuals can also experience immune dysregulation and lung damage. Following pathogen invasion within the lungs, neutrophils are recruited to the site of infection where they act as first responders, initiating the release of local and systemic cytokines such as IL-4, IL-6, IL-10, IL-8, IL-1β, TNF-α, and transforming growth factor (TGF)-β, a process which is largely dependant on the invading microbe [20,21]. However, if left unchecked, excessive inflammation can lead to severe disease, tissue remodeling, lung fibrosis, and pulmonary dysfunction [20,22].

A history of pneumonia has been listed as a risk factor for airway obstruction and although information remains limited, researchers have begun to associate pneumonia-causing agents with lung function decline [22–26]. In Ralph *et al*, airflow obstruction (FEV_1_ <60% predicted) was seen in approximately half of the individuals with tuberculosis, with only a 14.8% improvement in FEV_1_ following treatment [23]. Rhee *et al* showed that patients cured of tuberculosis had a mean FEV_1_ decline of 38 mL/year, rates that were similar to those seen among COPD patients without tuberculosis [24]. In addition, persistent *Chlamydophila pneumoniae* infection has also been linked to decreased FEV_1_ (6 mL/year) and FVC (7 mL/year) in women [25].

Looking at individuals who have both HIV and pneumonia, Nelsing *et al* noted persistent reduction in DLCO up to 9 months post pneumonia infection [27] while Morris and coworkers observed that HIV-infected individuals who had either *Pneumocystis jirovecii* or bacterial pneumonia had a permanent decrease in FEV_1_, FVC, and FEV_1_/FVC ratio [28]. Although the underlying mechanisms behind these decreases have yet to be elucidated, investigations have attempted to correlate immune markers with lung damage and poor clinical outcomes. In a recent publication by Wang *et al*, 8 biomarkers including markers of inflammation (soluble TNF receptor [sTNFR]-1, sTNFR-2, C-reactive protein [CRP]), coagulation (D-dimer), T cell activation (sCD27), interferon response (IP-10), monocyte and macrophage activation (sCD14), and fibrosis (hyaluronan) were found to be elevated among HIV and pneumonia co-infected individuals, even after adjusting for pneumonia severity [11]. Moreover, these cytokines were predictive of short-term mortality after pneumonia. Since tuberculosis is often the main cause of pneumonia among HIV patients, and IFN pathways have been reported as one of the most important pathways in tuberculosis [29], it would be interesting to study the role that this immune marker plays in lung abnormality in further studies.

Collectively, studies suggest a potential role for a variety of immune markers in lung dysfunction in individuals with HIV and/or pneumonia. Consequently, the aim of this systematic review was to provide an overview of inflammatory markers associated with lung abnormalities in HIV, pneumonia or HIV and pneumonia to gain insight into lung disease among HIV and pneumonia co-infected individuals and to suggest potential targets for anti-inflammatory treatment.

## Materials and methods

This systematic review was registered with the International Prospective Register of Systematic Reviews (PROSPERO) on August 15, 2017 (registration number CRD42017069254- S1 File).

### Search strategy and information sources

Following the Preferred Reporting Items for Systematic Reviews and Meta-Analyses (PRISMA) guidelines, we performed an exhaustive search using 4 databases: Cochrane Central Register of Controlled Trials, PubMed Central, Clinical Trials.gov and Google Scholar [30,31] (see S2 File). Search terms included the following MeSH terms and keywords: cytokines, chemokines, biomarkers, lung function, lung function decline, lung injury, lung inflammation, pneumonia, community-acquired pneumonia, and HIV.

Studies were included if they reported on: (i) adult populations (>18 years old); (ii) HIV, pneumonia (caused by any pathogen), or HIV with pneumonia; (iii) cytokines, chemokines, or biomarkers; (iv) lung abnormalities; and were (v) clinical trials, observational or comparative studies. The exposure of interest was cytokine exposure, while the study outcome was lung abnormality (not mortality which could be attributable to a variety of reasons). Lung abnormality includes structural and functional abnormalities and was defined using lung function tests, diffusion capacity, or imaging (looking for emphysema or fibrosis). For cohort and clinical trials, we verified that the paper described that the outcome was not present at the time of enrolment. The main covariates of interest were HIV serostatus and the presence of infectious pneumonia. Studies that focused on patients with lung cancer, cystic fibrosis, COPD, hospital-acquired pneumonia, and acute respiratory distress syndrome (ARDS) were excluded. Non-HIV causes of immunosuppression (organ transplant, autoimmune disorders, neutropenia) were also excluded, as were descriptive studies, case reports and series, reviews and conference abstracts.

For Cochrane, PubMed, and Clinical Trials, all studies published before June 2018 were eligible for review. For Google Scholar, due to the extensive number of abstracts that were returned during our search, only studies published after 2014 were sought. There were no setting restrictions, however, only articles published in English were reviewed.

### Study selection and data extraction

To identify which studies met our inclusion criteria, abstracts/summaries retrieved during the search strategy were screened independently by 2 reviewers (BMH and RM). All disagreements regarding study eligibility were resolved using a third reviewer. The full text of each of the identified studies was then assessed for eligibility. A pre-determined extraction table was used for data extraction and quality assessment (S3 File). Extracted information included: year of publication, the country in which the research took place, study design (cohort, clinical or cross-sectional), population, duration of follow-up, total number and age of participants, techniques and samples used for cytokine measurement, etiology of lung disease, cytokines which were deemed significant, smoking and HIV history (whether on cART, if/when diagnosed, CD4+ T cell count, viral load), and any previous hospitalization for lung infection/disease.

To ensure completeness of our review, a manual search on all of the references from each screened article was also conducted.

### Assessment of risk of bias

Both reviewers independently assessed the risk of bias of the exposure, outcome, and comparisons of each included study using previously established quality assessment scales (the Jadad and the Newcastle-Ottawa Quality Assessment Scales and the National Institutes of Health Cross-Sectional Quality Assessment Tool) [32–34]. Cohort studies were evaluated based on selection (representativeness of the cohort, ascertainment of exposed individuals, demonstration that the outcome was not present at study initiation), comparability (cohorts are comparable in design or analysis), and outcome (assessment of the outcome and follow-up). Clinical trials were assessed for randomization (whether mentioned and appropriate), concealment, blinding (whether mentioned and appropriate), and patient accountability (all patients accounted for including withdrawals and dropouts). Lastly, cross-sectional studies were evaluated based on their research question, objective and population (whether specified and well-defined); selection (representativeness of the population), participation rate, blinding (whether mentioned and appropriate), exposure and outcome (well defined, reliable and consistent across the study participants), and statistical analyses (whether appropriate and if they controlled for confounding variables).

### Summary measures

Due to heterogeneity in study design and method of cytokine measurement between the identified studies, a qualitative synthesis was performed.

## Results

### Study selection

**Fig 1:**
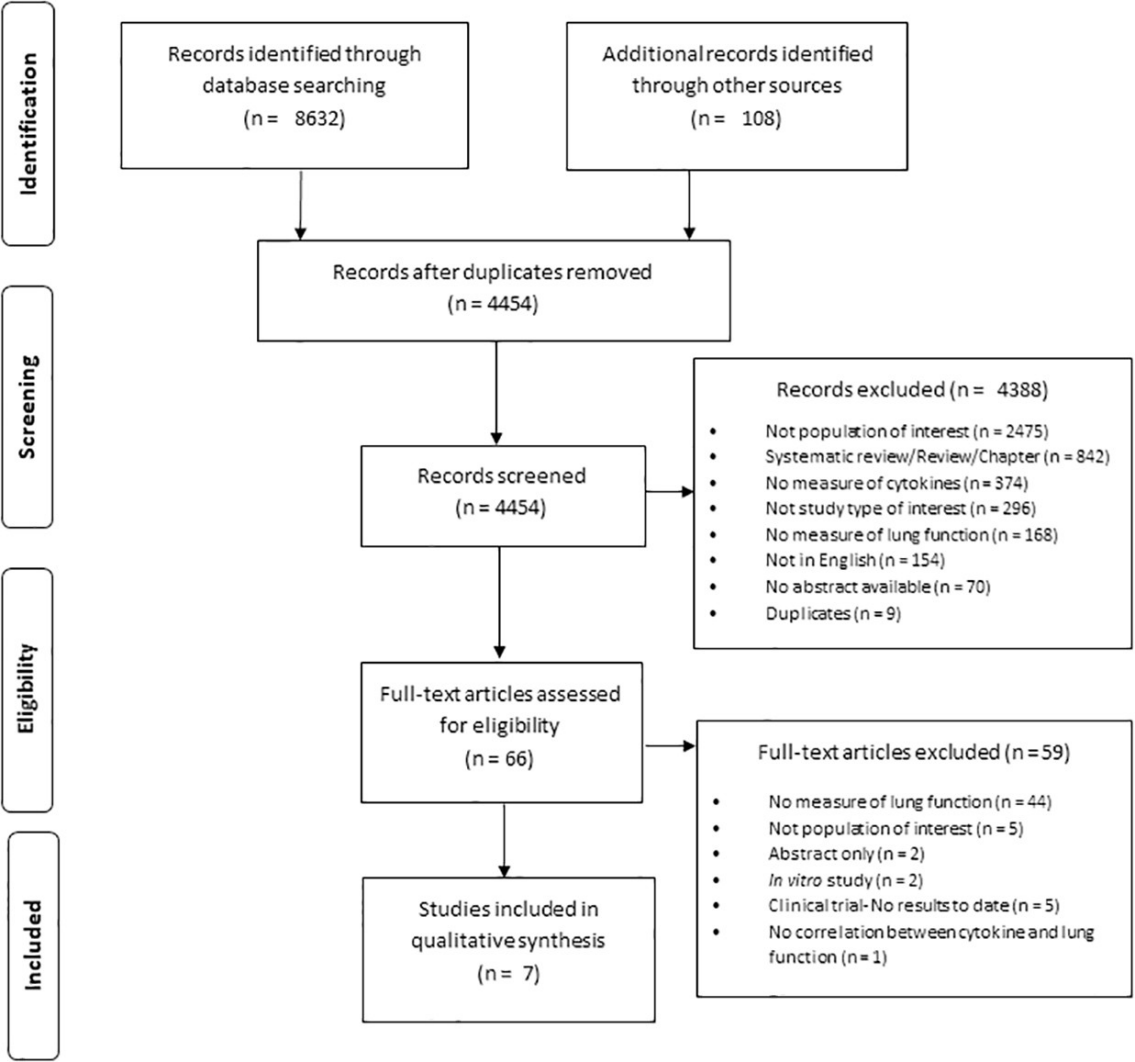
PRISMA flow diagram of the search and review process.

The search strategy identified 8632 records while the manual search identified an additional 108 records that had not appeared using the search strategy (Fig 1). After removing the duplicates, 4454 unique records remained. Of those, 66 full texts were assessed for eligibility from which 59 were excluded for not meeting our inclusion criteria (S3 File).

Seven articles met our criteria and were included for review [35–41]. The articles included 6 cross-sectional studies and 1 cohort study, all of which investigated the association between circulating biomarkers and lung abnormality among HIV-infected individuals. None of the included articles reported on patients with HIV and pneumonia nor pneumonia alone.

### Critical appraisal of the included studies

Following the risk of bias assessment, studies were scored based on high, moderate or low risk of bias due to methodological limitations (S4 File). In several of the included studies, sample size or power calculations are not included, limiting the external validity of their findings. Four of the included studies were at risk of observer bias due to the lack of reported blinding of the investigators, radiologists or interpreters [36,38–40]. In addition, several studies are at risk of sampling bias [35,36,40]. In North *et al*, groups were chosen by convenience sampling [40], while in Attia and Triplette [35,37], patients were chosen from a veteran population, which limits the generalizability of these results [35,37]. The study by Crothers *et al* is at risk of confounding since they had limited ability to adjust for multiple covariates and confounders within their groups [41]. Lastly, in Fitzpatrick *et al*, inclusion and exclusion criteria were not specified, therefore it is unknown whether the sample population is representative of the general population making their investigations at risk for selective reporting [38].

### Patient characteristics

Seven studies involving 1737 patients examined biomarkers and their association with lung structure or function (Table 1). Altogether, 1089 HIV-infected individuals and 648 controls were compared. Patients were primarily male, of black ethnicity, and were either current or former smokers.

**Table 1:** Demographic characteristics of patients from each included study.

	Attia 2014 [35]	Fitzpatrick 2014 [39]	Lambert 2014 [36]	Crothers 2016 [41]	Fitzpatrick 2016 [38]	Triplette 2017 [37]	North 2018 [40]
**Number of participants**	203	147	650	39	274	190	234
**Country**	USA	USA	USA	USA	USA	USA	Uganda
**Age (years)**	55(50–58)^b^	45.6(9.4)^a^	48.6(8.0)^a^	40–57^c^	54(48–61)^b^	55(49–59)^b^	52(48–55)^b^
**Sex (n, %)**							
Female	21(10)	49(33)	227(35)	-	0(0)	4(2)	107(46)
Male	182(90)	98(67)	423(65)	-	274(100)	186(98)	127(54)
**Ethnicity (n, %)**							
White	42(21)	-	N/A	-	213(78)	24(13)	-
Black	136(67)	82(56)	592(91)	30 (77)	47(17)	135(71)	234(100)
Other	22(11)^¶^	5(3.4)*	N/A	-	14(5)^¶^	31(16)^¶^	-
**Smoking status (n, %)**							
Current	46(23)	84(57.1)	556(85.5)	39(100)	-	120(63)	35(15)
Former	-	-	60(9.2)	-	-	40(21)	80(34)
Never	36(18)	25(17)	34(5.2)	-	-	29(15)	119(51)
**Smoking, pack years**	-	10(1.4–22)^b^	23.8(16.9)^a^	8–40^c^	1.9(0–22.1)^b^	3–42^c^	0(0–18)^b^

N/A: Not applicable;—not mentioned

^a^ mean (standard deviation)

^b^ median (interquartile range)

^c^ range

*Hispanic

^¶^Hispanic and other

### Immunological markers

Eleven biomarkers were analyzed in the 7 studies (see Table 2) with IL-6 and sCD14 assessed the most frequently (n = 5). Majority of the studies consisted of cross-sectional assessments and measured cytokines in blood (plasma or serum) using an enzyme-linked immunosorbent assay (ELISA).

**Table 2:** Characteristics and descriptions of the included articles.

Author	Study Type	Population (n)	Sample type	Immunological markers assessed/ Method	Lung function methodology
Attia 2014 [35]	Cross-sectional	HIV-positive (114) HIV-negative (49)	Blood (serum)	**IL-6, D-dimer, sCD14:** Immunoassay (NM)	PFT Radiograph
Fitzpatrick 2014 [39]	Cross-sectional	HIV-positive (147)	Blood (plasma)	**hsCRP**: ELISA (Phoenix Pharmaceuticals, Burlingame, CA, USA) **IL-6, IL8**: ELISA (R&D, Minneapolis, MN, USA)	PFT
Lambert 2014 [36]	Cross-sectional	HIV-positive (370) HIV-negative (280)	Blood (plasma)	**Cathelicidin**: ELISA (Hycult Biotech, Uden, Netherlands)	PFT
Crothers 2016 [41]	Cross-sectional	HIV-positive (19) HIV-negative (20)	Blood (plasma/serum*)	**IL-6:** Immunoassay (R&D, Minneapolis, MN, USA) **sCD14**: Immunoassay (R&D, Minneapolis, MN, USA)	PFT Radiograph
Fitzpatrick 2016 [38]	Cohort	HIV-positive (124) HIV-negative (150)	Blood (serum)	**IL-8, sCD14, sCD163, IL- 2sRa, ET-1**: ELISA (R&D, Minneapolis, MN, USA) **IL-6, TNFa:** Luminex (Luminex corporation, Texas, USA)	PFT
Triplette 2017 [37]	Cross-sectional	HIV-positive (190)	Blood (plasma)	**sCD14, CD4/CD8 ratio:** Flow cytometry	PFT Radiograph
North 2018 [40]	Cross-sectional	HIV-positive (125) HIV-negative (109)	Blood (serum- hsCRP; plasma- IL-6, sCD14, sCD163)	**hsCRP**: Latex immunoturbidimetry (LabCorp, Burlington, NC, USA) **IL-6:** ELISA (MesoScale Discovery, Rockville, MD, USA); **sCD14, sCD163:** ELISA (R&D, Minneapolis, MN, USA)	PFT

ELISA: Enzyme-linked immunosorbent assay, NM: Not mentioned; PFT: Pulmonary function test;

*Crothers *et al* [41] describes the use of serum IL-6 in their methods but report on plasma in their results

Nine biomarkers, including IL-6, TNF-α, CRP, cathelicidin, sCD14, sCD163, IL-2 receptor α chain (IL-2sRα), endothelin-1 (ET-1), and CD4/CD8 ratio had significant associations with lung abnormality, however findings were discordant as there was little overlap reported amongst the cytokines that had significant associations (Table 3). Nonetheless, cytokines can be grouped based on pathways, such as markers involved in general inflammation (IL-6, TNF-α, CRP), innate defense (cathelicidin), monocyte and macrophage activation (sCD14, sCD163 and, IL-2sRα), endothelial dysfunction (ET-1) or general biomarkers of immune system health (CD4/CD8 ratio). Below we summarize the results for each of the 9 cytokines.

**Table 3:** Study findings of the included articles, stratified by immune marker.

Immune marker	Author	Sample size with data	Immunological markers		Method of lung assessment	Associations between immunological markers and lung findings
			Assessment method	Findings		
**IL-6**	Attia 2014 [35]	203	Immunoassay	**Median (IQR), pg/mL:** *** HIV-positive: 1.81 (1.28–3.43) HIV-negative: 1.23 (0.94–2.07)	PFT Radiograph	No significant association between IL-6 and emphysema severity
	Crothers 2016 [41]	40	Immunoassay	**Median, pg/mL:** *** HIV-positive with low DLCO: 4 HIV-negative with low DLCO: < 2	PFT Radiograph	HIV+ subjects with low DLCO (≤60% predicted value) had higher IL-6 concentrations (4 and <2 pg/mL) ***
	North 2018 [40]	234	ELISA	**Results:** HIV-positive: higher (exact values NM) HIV-negative: lower (exact values NM)	PFT	HIV-infected individuals with IL-6 levels in the 4^th^ quartile, had lower FEV_1_ (-18.1 ml [95% CI -29.1, -7.1] and FVC (-17.1 [95% CI -28.2, -5.9]) ***
	Fitzpatrick 2014 [39]	123	ELISA	**Median (IQR), pg/mL:** HIV: 2.0 (1.1–3.3)	PFT	IL-6 negatively correlated with DLCO (r -0.075) and FEV_1_%-predicted (r -0.074) *** Individuals with elevated IL-6 had greater odds of impaired DLCO (<60% predicted values; OR 3.758) ***
	Fitzpatrick 2016 [38]	259	Luminex	**Median (IQR), pg/mL:** HIV-positive: 10.9 (1.7–21.1) HIV-negative: 7.0 (1.2–20.8)	PFT	IL-6 associated with worse FEV_1_%-predicted and DLCO values among HIV-infected participants***
**TNF-α**	Fitzpatrick 2016 [38]	260	Luminex	**Median (IQR), pg/mL:** HIV-positive: 33.4 (5.0–69.4) HIV-negative: 35.9 (3.8–62.0)	PFT	TNF-α associated with lower DLCO%-predicted values in HIV-infected individuals with similar trends seen among the control group***
**CRP**	North 2018 [40]	234	Latex immunoturbidimetry	**Results:** *** HIV-positive: higher (exact values NM) HIV-negative: lower (exact values NM)	PFT	HIV-infected individuals that had CRP >3 mg/L had lower FEV_1_ (-39.3mL [95% CI -61.7, -16.9]) and FVC (-44 mL, [95% CI -48.4, -6.4]) compared to individuals with CRP levels <3 mg/L ***
	Fitzpatrick 2014 [39]	123	ELISA	**Median (IQR), mg/L:** HIV: 1.6 (0.1–6.8)	PFT	CRP (>1 mg/L) associated with FEV_1_% predicated and DLCO *** CRP (>1 mg/L) associated with greater odds of impaired DLCO (OR 3.758) ***
**Cathelicidin**	Lambert 2014 [36]	650	ELISA	**Median (IQR), ng/mL:** HIV-positive: 35.5 (28.4–44.6) HIV-negative: 36.4 (29.2–47.0)	PFT	Low cathelicidin (< 28.8 ng/mL) associated with decreased levels of FEV_1_ (-115 mL [95% CI -221, -8]) ***
**sCD14**	Attia 2014 [35]	203	Immunoassay	**Median (IQR), ng/mL:** *** HIV-positive: 1671 (1472–2128) HIV-negative: 1386 (1171–1569)	PFT Radiograph	High sCD14 (>1883 ng/mL) associated with increased (>10%) emphysema *** HIV-infected individuals with nadir CD4+ T cell count <200 cells/mL had increased odds of emphysema (OR 2.39, 95% CI 1.02, 5.62) ***
	Crothers 2016 [41]	40	Immunoassay	**Mean, ng/mL:** *** HIV-positive: 1681 HIV-negative: 1367	PFT Radiograph	No significant association between sCD14 and DLCO status
	Triplette 2017 [37]	190	Flow cytometry	**Median (IQR), ng/mL:** HIV-positive: 1674 (1487–2164) HIV-negative: 1626 (1364–1953)	PFT Radiograph	No significant association between sCD14 and emphysema severity or decreased lung function
	Fitzpatrick 2016 [38]	263	ELISA	**Median (IQR), pg/mL:** *** HIV-positive: 2128.6 (1831.2–2522.9) HIV-negative: 1782.5 (1607.2–2075.3)	PFT	No significant association between sCD14 and lung abnormalities in either group
	North 2018 [40]	234	ELISA	**Results:** *** HIV-positive: higher (exact values NM) HIV-negative: lower (exact values NM)	PFT	No significant association between sCD14 and lung function
**sCD163**	North 2018 [40]	234	ELISA	**Results:** HIV-positive: higher (exact values NM) HIV-negative: lower (exact values NM)	PFT	In the HIV group, sCD163 associated with lower FVC (-14.3 ml [95% CI -26.9 to -1.7]) ***
	Fitzpatrick 2016 [38]	274	ELISA	**Median (IQR), ng/mL:** *** HIV-positive: 862.5 (640.1–1099.9) HIV-negative: 649.5 (499.2–847.0)	PFT	sCD163 associated with lower FEV_1_/FVC ratios and lower DLCO %-predicted ***
**IL-2sRα**	Fitzpatrick 2016 [38]	274	ELISA	**Median (IQR), pg/mL:** *** HIV-positive: 942.9 (694.6–1256.9) HIV-negative: 814.7 (646.0–1022.0)	PFT	In both populations, IL-2sRα associated with lower DLCO%-predicted***
**ET-1**	Fitzpatrick 2016 [38]	268	ELISA	**Median (IQR), pg/mL:** HIV-positive: 1.4 (1.1–1.7) HIV-negative: 1.3 (1.3–1.6)	PFT	ET-1 associated with lower FEV_1_%-predicted and increased odds of reduced FEV_1_/FVC ratios (OR 4.8) among HIV-infected individuals *** ET-1 associated with lower FEV_1_%-predicted over time in HIV-infected individuals ***
**CD4/CD8 Ratio**	Attia 2014 [35]	190	Flow cytometry	**CD4/CD8 ratio** < 0.4 **CD4/CD8 ratio** 0.4–1.0 **CD4/CD8 ratio** > 1.0	PFT Radiograph	CD4/CD8 ratio <0.4 associated with increased odds of having >10% emphysema compared to those with a CD4/CD8 ratio >1.0 (OR 7.4; 95% CI 1.5, 35) *** Patients with low CD4/CD8 ratio (<0.4) had reduced DLCO% (51 [IQR 41–58] vs 59 [IQR 49–71]) ***

ELISA: Enzyme-linked immunosorbent assay; FEV_1_: Forced expiratory volume in 1 second; FVC: Forced vital capacity; DLCO% predicted: % predicted of carbon monoxide diffusion capacity; NM: Not mentioned; PFT: Pulmonary function test; r: correlation coefficient

*** indicates a significant result (*P*≤0.05).

#### Markers of inflammation (IL-6, TNF-α, and CRP)

Five studies looked at the relationship between IL-6 and lung abnormality among HIV-infected populations [35,38–41]. In each of the studies, IL-6 concentrations were higher among people living with HIV and in 4 of the studies, increased IL-6 associated with decreased lung function [38–41]. Crothers *et al* [41] reported that HIV patients that had low DLCO (≤60% predicted value) had plasma IL-6 concentrations twice that of those with preserved DLCO (4 and <2 pg/mL, respectively, *P*<0.005). Similarly, Fitzpatrick 2014 [39] found that plasma IL-6 concentrations correlated negatively with DLCO and FEV_1_%-predicted (correlation coefficients of -0.075, *P* = 0.005 and -0.074, *P* = 0.03, respectively). In addition, individuals that had IL-6 levels >3.3 pg/mL had 3.7 times greater odds of having DLCO% predicted <60 (*P* = 0.006). In keeping with these findings, Fitzpatrick 2016 *et al* [38] reported that IL-6 associated with significantly worse FEV_1_%-predicted and DLCO values among the HIV-infected participants. North *et al* also reported that per IQR increase in IL-6 in individuals with HIV, FEV_1_ and FVC saw a significant decrease by 18.1 ml (95% CI -29.1, -7.1) and 17.1 (95% CI -28.2, -5.9), respectively [40]. In contrast, studies by Attia *et al* reported no significant association between IL-6 and emphysema severity.

Only one study looked at TNF-α and its association with lung function decline [38]. In Fitzpatrick 2016 *et al*, TNF-α had a significant association with lower DLCO%-predicted values in HIV-infected individuals, with similar trends also seen among the uninfected controls.

Two studies assessed the relationship between CRP and lung dysfunction in the HIV-infected population, both of which reported higher CRP levels among HIV-infected individuals [39,40]. Fitzpatrick *et al* found that plasma CRP (>1 mg/L) associated with significantly lower FEV1%-predicted values and higher odds (2.5 times) of airflow obstruction (FEV_1_/FVC ratio <0.7, OR 2.545, *P* = 0.06) [39]. In addition, individuals who had elevated CRP (>1 mg/L) had lower DLCO (*P* = 0.001) and had 3.7 times greater odds of having impaired DLCO (*P* = 0.006). In *post-hoc* analyses controlling for undetectable HIV viral load, elevated CRP retained an association with worse DLCO.

In the second study, median CRP concentrations were higher among individuals with HIV compared to uninfected controls (*P*<0.001) however, elevated CRP (>3 mg/L) associated with reduced FEV_1_ and FVC in both patient groups [40]. HIV-infected individuals that had elevated CRP had a 39.3 mL reduction in FEV_1_ (95% CI -61.7, -16.9) and a 44 ml decrease in FVC (95% CI -48.4, -6.4). Uninfected controls exhibited similar results (FEV_1_: 37.9 ml [95% CI -63.2, -12.6]; and FVC 58.0 ml [95% CI -88.4, -27.5]).

#### Innate antimicrobial peptides (cathelicidin)

Cathelicidin was the only peptide that was investigated for its role in lung disease among our study populations. In Lambert *et al* [36], no association between HIV serostatus and median cathelicidin levels was observed (*P* = 0.12), however, individuals with low cathelicidin (<28.8 ng/mL) demonstrated a 115 mL decrease in FEV_1_ (95% CI -221, -8; *P* = 0.035) compared to individuals with high cathelicidin.

#### Markers of monocyte activation (sCD14, sCD163 and IL-2sRα)

Five studies measured sCD14 in HIV-infected individuals, 4 of which found no significant association between sCD14 and lung abnormality [35,37,38,40,41]. Crothers and colleagues reported significantly higher concentrations of sCD14 in HIV patients (1681 vs. 1367 ng/mL), however, their populations did not differ by DLCO status [41]. Triplette and coworkers write that median sCD14 concentrations were similar among individuals with HIV and controls (1674, IQR 1487–2164; vs 1626, IQR 1364–1953 ng/mL; *P* = 0.13) and did not associate with decreased lung function nor disease severity [37], a view which was also supported by North [40] and Fitzpatrick [38]. In contrast, Attia *et al* [35] observed that HIV-infected individuals had significantly higher median serum sCD14 concentrations compared to controls (1671, IQR 1472–2128; vs 1386, IQR 1171–1569 ng/mL) and that individuals who had >10% emphysema had higher sCD14 compared to those with <10% emphysema (1883 vs 1648 ng/mL; *P*<0.05). Additionally, they found that patients with a nadir CD4+ T cell count <200 cells/mL had 2 times greater odds of having increased emphysema (OR 2.39; 95% CI 1.02, 5.62).

Two studies looked at the association between sCD163 and lung abnormalities in the HIV-infected population, both of which reported decreased lung function among individuals with elevated sCD163 [38,40]. North *et al* found that although median sCD163 levels were similar among study populations, sCD163 associated with a 14.3 ml decrease in FVC (95% CI -26.9, -1.7) in the HIV group. Fitzpatrick and colleagues come to similar conclusions in their HIV population, stating that elevated sCD163 associated with significantly lower FEV_1_/FVC ratios and DLCO during follow-up [38].

Only 1 study, Fitzpatrick 2016, reported on IL-2sRα in lung function studies among HIV-infected individuals [38]. In their analysis, investigators found that IL-2sRα concentrations were significantly higher among those with HIV (942.9, IQR 694.6–1256.9; vs 814.7, IQR 646.0–1022.0 pg/mL) however, in both populations, IL-2sRα associated with lower DLCO%-predicted (*P* = 0.005 and *P* = 0.004). Interestingly, this association was lost over time.

#### Markers of endothelial dysfunction (ET-1)

Fitzpatrick et al [38] was the only study that investigated the association between ET-1 and lung abnormality among the HIV-infected population. ET-1 concentrations were similar among the HIV group and the uninfected controls (1.4, IQR 1.1–1.7; and 1.3, IQR 1.3–1.6 pg/mL, respectively; *P* = 0.4), however HIV-infected individuals who had higher ET-1 had significantly lower FEV_1_%-predicted values, and had 4.8 greater odds of having lower FEV_1_/FVC ratios (OR 4.8, *P* = 0.04) compared to controls.

When looking at the relationship between ET-1 and lung function over time, baseline levels of ET-1 associated with decreased FEV_1_%-predicted, and DLCO in HIV-infected individuals [38]. However, ET-1 was not able to predict a greater rate of decline of FEV_1_ or DLCO.

#### Markers of general immune health (CD4/CD8 ratio)

One study which set out to determine the relationship between plasma CD4/CD8 ratio and lung abnormality found that low CD4/CD8 ratio (<0.4) associated with increased emphysema (>10% involvement) [37]. After adjusting for CD4 count and HIV viral load, individuals with a CD4/CD8 ratio <0.4 had 7.4 times the odds of >10% emphysema compared to those with a CD4/CD8 ratio >1.0 (OR 7.4, 95% CI 1.5, 35). Patients with low CD4/CD8 ratio also had reduced DLCO% (51, IQR 41–58; vs 59, IQR 49–71 for low and high CD4/CD8 ratio, respectively; *P* = 0.008).

## Discussion

The present review assessed cytokines associated with lung abnormalities among HIV and/or pneumonia-infected individuals. Seven studies [35–41] were identified in our search from which 11 cytokines were studied for their association with lung abnormalities among HIV-infected individuals. Interestingly, none of the articles reported on biomarkers and lung structure or function among individuals with HIV and pneumonia co-infection, nor pneumonia alone, reflecting a gap in knowledge regarding the pathogenesis of lung disease among these populations.

Although 9 immune markers (IL-6, TNF-α, CRP, cathelicidin, sCD14, sCD163, IL-2sRα, ET-1, and CD4/CD8 ratio) associated with decreased lung function or lung abnormalities, there was discordance in markers studied and differences identified between the studies. For example, of the 5 studies that reported on IL-6, Attia *et al* did not find a significant association between IL-6 and lung abnormality among their HIV-infected population [35]. However, more prevalent smoking has been associated with both IL-6 and lower lung function [42] and as Attia *et al* reported a lower percentage of cigarette smokers among their study cohort, this may have contributed to their result. Attia *et al* also reported a relationship between sCD14 and emphysema with greater odds of emphysema among their HIV patients, results that differ from those seen in the other 4 studies which looked at sCD14 [37,38,40,43]. This divergence could be attributable to the fact that Attia *et al* measured biomarkers in serum while the others looked at plasma since detectability and measurability differences between serum and plasma have been documented [44]. Conversely, different methods of immunological assessment that have different sensitivity levels may also be a contributing factor. Another potential and very important confounder that many publications did not report, is cART and CD4 count, both of which could reflect different stages of immunosuppression and could affect cytokine production. In addition, HIV patients often have multiple coinfections, for example, sexually transmitted blood-borne infections, which can affect cytokines results however, many of the included studies did not mention this information.

Nonetheless, similar trends among the studies were that people living with HIV have higher concentrations of IL-6, CRP, sCD14, sCD163, IL-2sRα and ET-1 and lower concentrations of cathelicidin and CD4/CD8 ratio, with similar concentrations of TNF-α seen in HIV-positive individuals and controls, and that IL-6, TNF-α, CRP, cathelicidin, sCD14, sCD163, IL-2sRα, ET-1, and CD4/CD8 ratio associated with lung abnormalities. Since several of these cytokines represent similar pathways, i.e. inflammation, innate defense, macrophage activation, endothelial dysfunction, and general immune health, this discussion will focus on candidate pathways of inflammation and/or immune activation, how these biomarkers relate to one another and how their presence in HIV and pneumonia co-infected individuals may lead to increased lung abnormality.

Within a normal and healthy lung, the innate and adaptive immune systems work together to maintain a homeostatic state. The airway epithelium and residential macrophages serve as the first-lines of defense, able to sense the environment and react to external dangers and cues through initiation or suppression of the adaptive immune response [45]. If no danger is present, the immune system remains immune quiescent [45,46]. However, in the case of HIV, immune activation and improper regulation of the immune network are a hallmark of disease. Systemic and local inflammatory markers such as TNF-α, IL-6, and CRP have been shown to be overexpressed in HIV infection, pneumonia and other diseases [5,21,47–49], with Fitzpatrick *et al* 2016 reporting a strong association between IL-6 and TNF-α (r = 0.87) [38]. High concentrations of TNF-α can cause localized cell damage and epithelial channel disruption leading to barrier dysfunction within the lungs [47]. Moreover, TNF-α, along with ET-1, can cause eosinophilic recruitment and fibrinogenic cytokine production, all of which can contribute to lung damage [50,51]. Although IL-6 and TNF-α can be produced by fibroblasts, lymphoid, and endothelial cells, monocytes and macrophages are their main source [52] thus, it is not surprising that markers of monocyte and macrophage activation, such as sCD14, sCD163, and IL-2sRα, were also elevated in Fitzpatrick *et al* [38]. sCD14, sCD163, and IL-2sRα are biomarkers that aid in pathogen surveillance and phagocytosis, inducing immune activation and amplification of subsequent host responses [53–55]. Among HIV-infected individuals, moderate associations between and sCD163 and IL-2sRα have been reported [38]. Increased activation of these markers and their downstream effects is what we hypothesize led to the increased emphysema seen in Attia *et al* [35]. sCD14 responses, although beneficial, can affect the lung through excessive inflammation and pathogen dissemination. sCD14 present in the bronchoalveolar space of *Streptococcus pneumoniae*-infected mice was shown to cause invasive pneumonia [56]. In serum of HIV-infected individuals, elevated sCD14 associated with the occurrence of immune activation-induced end-organ dysfunction and poor prognosis despite cART [57–59]. Similar results in HIV patients and in individuals with pneumonia have also been seen with sCD163 [38,60].

An association between low cathelicidin levels and history of bacterial pneumonia has also been observed [36]. Cathelicidin, an innate response protein that acts as a cell signaling and chemotaxis molecule, serves as a natural antimicrobial against viruses, bacteria and fungi [61]. Although it can be expressed by numerous cell types (macrophage, mast cells, and airway epithelial cells), it is perpetually available within neutrophils [62–64]. Upon stimulation of the exterior of the neutrophil, cathelicidin is released into the extracellular milieu triggering a cascade of events including induction of incoming neutrophils, recruitment of monocytes, eosinophils, mast cells and T lymphocytes and initiation of epithelial regeneration and remodeling [61,62,65]. High concentrations of cathelicidin can contribute to epithelial injury and destruction through overstimulation and overproduction of structural cells [62]. Conversely, at low levels, it was also correlated with lung dysfunction among individuals with HIV [36]. Although these findings are contradictory and need to be explored further, cathelicidin may play a role in lung disease.

**Fig 2:**
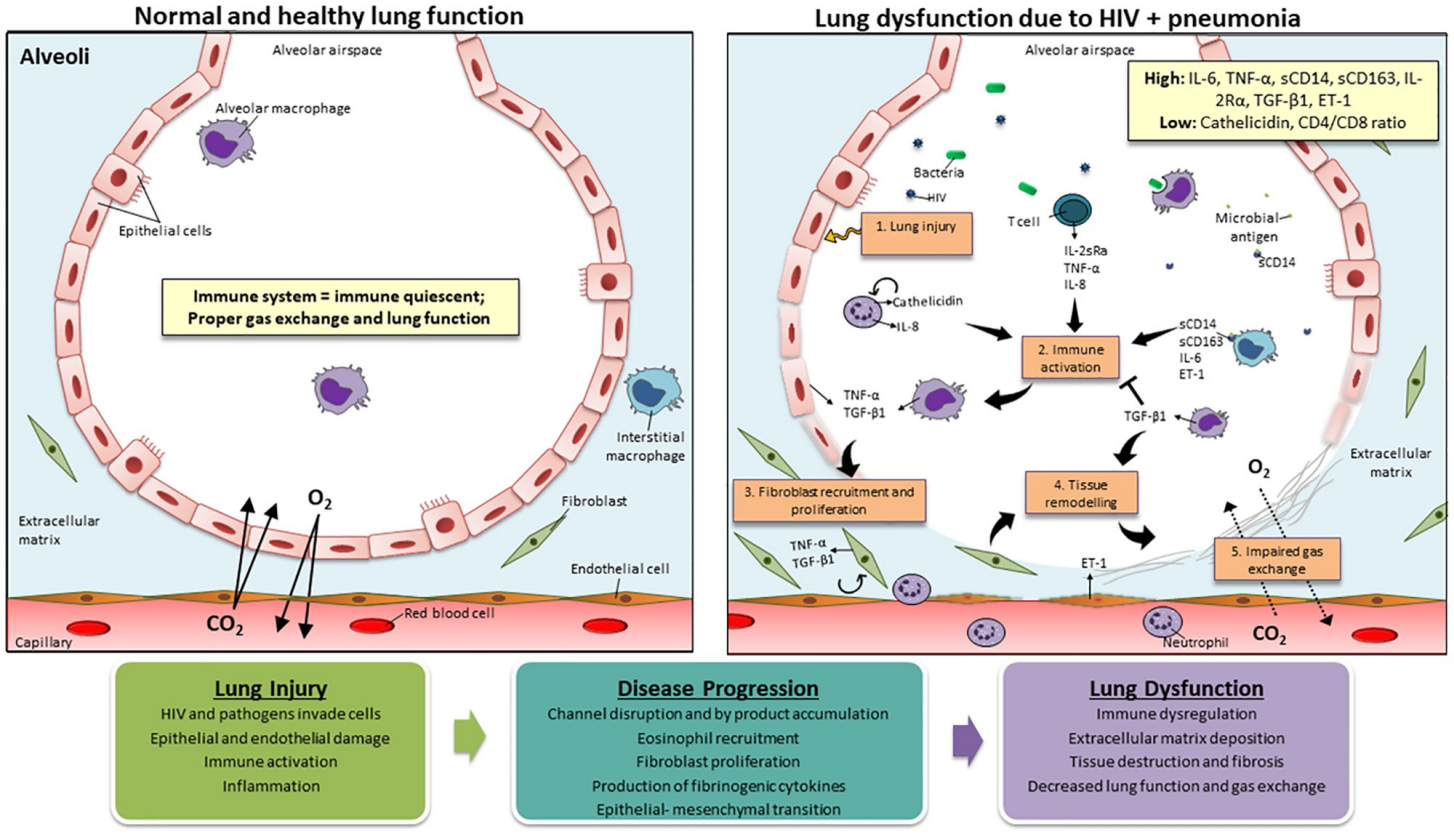
A proposed framework for the hypothesized pathogenesis of lung abnormality among individuals with HIV and pneumonia co-infection. In a normal and healthy alveolus, the immune system works together to maintain an immune quiescent state (top left panel) [45]. However, in individuals with HIV and in those with pneumonia, innate response markers are affected [36,63] and elevated levels of markers of inflammation (IL-6, TNF-α and CRP) [5,21,35,40,41,48,49,68,69], macrophage activation (sCD14, sCD163, and IL-2sRα) [35,37,40,41,55,66,69] and endothelial dysfunction (ET-1) [69] have been reported. HIV alters the immune environment and affects the host response [67], therefore it is hypothesized that HIV-infected individuals who contract pneumonia will have a decreased ability to clear the invading pathogen, leading to increased fibroblast activation, extracellular matrix deposition and tissue remodeling resulting in impaired gas exchange and greater lung abnormality (top right panel).

Elevated levels of markers of inflammation, and monocyte and macrophage activation have been documented in both individuals with HIV and individuals with pneumonia [5,8,21,38,40,47–49,55,66]. Likewise, innate response markers are also affected [36,63]. Since HIV alters the immune environment and affects alveolar macrophage response to bacteria [67], it is logical to think that HIV-infected individuals who contract pneumonia will have a decreased ability to clear the invading pathogen, leading to increased damage and lung abnormality. A proposed framework for the hypothesized pathogenesis of lung abnormality among individuals with HIV and pneumonia co-infection can be seen in Fig 2.

Although this review identified cytokines associated with lung abnormalities and found more lung function decline among people living with HIV compared to uninfected controls, it could not identify a causal relationship between these factors. There may be several pathways to the low pulmonary function observed in the HIV-infected population. Li *et al* examined the progression of pulmonary function measurements in an HIV cohort over a 6-year period [70]. They found that a history of pneumonia was significantly associated with DLCO% decline. Similar results were also reported by Gupte and colleagues who found that prior TB infection associated with an excess loss of FEV_1_, and that although older age, smoking and higher CRP associated with obstructive lung disease, CD4 count and ART did not [71]. Similarly, Kunisaki and coworkers found no difference in lung dysfunction between HIV patients on early or deferred cART in their study which followed patients for 2 years [72]. Likewise, in one of the largest cohorts which looked at pulmonary function in people living with HIV and uninfected controls, Ronit *et al* found that HIV was a risk factor for decreased FEV_1_ and FVC despite being on cART (n = 1064, 98.5%) and having a viral load <50 copies/mL (n = 1015, 94.6%) [12]. Similar findings were also reported in a cynomolgus macaques model which used simian-adapted HIV [73]. In contrast to expert opinion, these studies suggest that cART does not prevent lung dysfunction nor does it have much of an impact on lung function in HIV. Although most agree that there is not enough data on ART and its relationship with lung function, this hypothesis is intriguing and hints that other factors might be at play. Perhaps it is not the virus itself but rather the associated systemic immune activation, microbial translocation or dysbiosis. Studies aimed at better understanding lung dysfunction in the HIV-infected population are warranted and will be required to determine the role that ART, the virus, the microbiome, and inflammation have on lung function decline.

In this review, we could not do a quantitative analysis of the data due to heterogeneity in the methodologies used for quantifying cytokines (immunoassays, ELISA, Luminex, and flow cytometry) and lung abnormality (PFT and radiograph) and since many of the included publications relied on different statistical outputs, such as the correlation coefficient, odds ratio, percent predicted, confidence intervals, *P* values etc.. Additionally, each of the inflammatory markers were measured in blood, however circulating cytokines can be confounded by coinfections, and also may not truly reflect what is occurring locally in the lungs. Lastly, cytokine concentrations vary during infection which may potentially affect the interpretation of the results depending on when samples are collected [74–76]. Consequently, cross-sectional studies, like the ones identified herein which only sample at a single time point, are less desirable to evaluate causality than studies that sample repeatedly over time. Ideally, simultaneous sampling of blood and lung, in a prospective and longitudinal study may provide a better ability to understand the processes leading to lung abnormalities.

We are also aware that there are limitations at the review level. Since there were a small number of scientific publications available for this topic, we, unfortunately, were not able to find any information pertaining to lung dysfunction in HIV and pneumonia co-infected individuals nor individuals with pneumonia alone, highlighting the importance of having new studies to evaluate our hypothesis.

## Conclusions

The immune response and its role in lung disease is complex. This systematic review identified several biomarkers that were elevated among HIV-infected individuals with lung abnormality, however, the cross-sectional data preclude linking these biomarkers as the cause of the associated lung disease. This review highlights the lack of information available on the association of cytokines and lung disease in the adult HIV and pneumonia populations, a finding which reflects an opportunity for further research. Studying markers of immune activation and how cytokine profiles impact lung structure and function in HIV and pneumonia is important as it may provide candidate biomarkers for future studies to ascertain their predictive utility and may lead to potential targets for immunomodulatory intervention thereby improving patient outcomes for individuals with these types of coinfections.

## Supporting information

S1 File PROSPERO protocol. (PDF)

S2 File Search strategy. (DOCX)

S3 File Data extraction tables. (XLSX)

S4 File Study quality assessment. (XLSX)

S5 File PRISMA checklist. (DOC)
