# Supplementary material for: Inflammatory mediators and lung abnormalities in HIV: A systematic review
Source: PLoS One. 2019 Dec 12;14(12):e0226347. doi: 10.1371/journal.pone.0226347 (PMC6907827; doi:10.1371/journal.pone.0226347)
Supplement: S1 File — (PDF) [file pone.0226347.s001.pdf]

PROSPERO International prospective register of systematic reviews

Review title and timescale

1 Review title
Give the working title of the review. This must be in English. Ideally it should state succinctly the interventions or exposures being reviewed and the associated health or social problem being addressed in the review.
Inflammatory mediators of lung dysfunction in adults infected with pneumonia, HIV, or HIV and pneumonia: a systematic review

2 Original language title
For reviews in languages other than English, this field should be used to enter the title in the language of the review. This will be displayed together with the English language title.
N/A

3 Anticipated or actual start date
Give the date when the systematic review commenced, or is expected to commence.
12/06/2017

4 Anticipated completion date
Give the date by which the review is expected to be completed.
16/10/2017

5 Stage of review at time of this submission
Indicate the stage of progress of the review by ticking the relevant boxes. Reviews that have progressed beyond the point of completing data extraction at the time of initial registration are not eligible for inclusion in PROSPERO. This field should be updated when any amendments are made to a published record.

The review has not yet started ×

Review stage	Started	Completed
Preliminary searches	Yes	Yes
Piloting of the study selection process	Yes	Yes
Formal screening of search results against eligibility criteria	Yes	Yes
Data extraction	No	No
Risk of bias (quality) assessment	No	No
Data analysis	No	No

Provide any other relevant information about the stage of the review here.

Review team details

6 Named contact
The named contact acts as the guarantor for the accuracy of the information presented in the register record.
Zulma Rueda

7 Named contact email
Enter the electronic mail address of the named contact.
zulma.rueda@upb.edu.co

8 Named contact address
Enter the full postal address for the named contact.
Área de Investigación Escuela de Ciencias de la Salud Calle 78B # 72A-109 Medellin 05001000 Colombia

9 Named contact phone number
Enter the telephone number for the named contact, including international dialing code.
57(4)4488388 ext 19323

10 Organisational affiliation of the review
Full title of the organisational affiliations for this review, and website address if available. This field may be completed

as 'None' if the review is not affiliated to any organisation.
University of Manitoba and Universidad Pontificia Bolivariana

Website address:

11 Review team members and their organisational affiliations
Give the title, first name and last name of all members of the team working directly on the review. Give the organisational affiliations of each member of the review team.

Title	First name	Last name	Affiliation
Miss	Breanne	Head	University of Manitoba
Mr	Ruochen	Mao	University of Manitoba
Dr	Zulma	Rueda	Universidad Pontificia Bolivariana
Dr	Yoav	Keynan	University of Manitoba

12 Funding sources/sponsors
Give details of the individuals, organizations, groups or other legal entities who take responsibility for initiating, managing, sponsoring and/or financing the review. Any unique identification numbers assigned to the review by the individuals or bodies listed should be included.
N/A

13 Conflicts of interest
List any conditions that could lead to actual or perceived undue influence on judgements concerning the main topic investigated in the review.
Are there any actual or potential conflicts of interest?
None known

14 Collaborators
Give the name, affiliation and role of any individuals or organisations who are working on the review but who are not listed as review team members.

Title	First name	Last name	Organisation details

Review methods

15 Review question(s)
State the question(s) to be addressed / review objectives. Please complete a separate box for each question.
How does the cytokine profile impact lung function in individuals (> 18 years old) infected with HIV, pneumonia, or HIV and pneumonia

16 Searches
Give details of the sources to be searched, and any restrictions (e.g. language or publication period). The full search strategy is not required, but may be supplied as a link or attachment.
For this study, we searched 4 electronic databases which included PubMed, The Cochrane Library (Cochrane Central Register of Controlled Trials [CENTRAL]), Clinical Trials.gov and Google Scholar. The search strategy included only the agreed upon terms listed in the intervention: cytokines, chemokines, biomarkers, lung function, lung function decline, lung injury, lung inflammation, pneumonia, community-acquired pneumonia, HIV as well as a combination of these terms. The terms were combined with filters which selected for clinical, observational, and comparative studies targeting adult (>18 years old) populations. The literature search was restricted to english only. For CENTRAL, PubMed and Clinical Trials, all studies published until the search date were eligible for this review. For Google Scholar, only the studies published after 2014 were sought.

17 URL to search strategy
If you have one, give the link to your search strategy here. Alternatively you can e-mail this to PROSPERO and we will store and link to it.
https://www.crd.york.ac.uk/PROSPEROFILES/69254_STRATEGY_20170708.pdf

I give permission for this file to be made publicly available
Yes

18 Condition or domain being studied

Give a short description of the disease, condition or healthcare domain being studied. This could include health and wellbeing outcomes.

Lung dysfunction. Assessing the progressive loss of lung function in patients with HIV and pneumonia.

19 Participants/population

Give summary criteria for the participants or populations being studied by the review. The preferred format includes details of both inclusion and exclusion criteria.

Adults older than 18 years old

20 Intervention(s), exposure(s)

Give full and clear descriptions of the nature of the interventions or the exposures to be reviewed

Cytokines, chemokines or biomarkers in individuals with HIV and pneumonia. Cytokine. A broad group of host secreted proteins that are important for cell signaling. Chemokine. A chemotactic family of cytokines. Biomarkers. A substance secreted from the host that is indicative of an infection, disease or environmental exposure. HIV and pneumonia. A group of HIV-infected individuals who present with less than 1 month of respiratory symptoms and have been diagnosed with pneumonia.

21 Comparator(s)/control

Where relevant, give details of the alternatives against which the main subject/topic of the review will be compared (e.g. another intervention or a non-exposed control group).

Cytokines, chemokines or biomarkers in individuals with only HIV or pneumonia HIV. A group of individuals who are infected with the human immunodeficiency virus. Pneumonia. A group of individuals who have had respiratory symptoms for less than 1 month and have been diagnosed to have pneumonia. Healthy controls. A group of individuals who do not have HIV nor any other lung infection/ disease.

22 Types of study to be included

Give details of the study designs to be included in the review. If there are no restrictions on the types of study design eligible for inclusion, this should be stated.

We included clinical trials as well as clinical, observational and comparative studies in order to assess the effect of the inflammatory response on lung dysfunction between groups.

23 Context

Give summary details of the setting and other relevant characteristics which help define the inclusion or exclusion criteria.

All studies must measure cytokines and lung function.

24 Primary outcome(s)

Give the most important outcomes.

Lung dysfunction.

Give information on timing and effect measures, as appropriate.

Measured using a pulmonary function test or through thoracic imaging.

25 Secondary outcomes

List any additional outcomes that will be addressed. If there are no secondary outcomes enter None.

N/A

Give information on timing and effect measures, as appropriate.

N/A

26 Data extraction (selection and coding)

Give the procedure for selecting studies for the review and extracting data, including the number of researchers involved and how discrepancies will be resolved. List the data to be extracted.

Abstracts and summaries retrieved from the search strategy were screened independently by two reviewers in order to identify the potential studies which met the inclusion criteria. All disagreements regarding the eligibility of particular studies will be resolved using a third reviewer. The full text of all identified studies will then be retrieved and independently assessed for eligibility by both review members. We will evaluate the concordance between the two reviewers using intraclass correlation coefficient and kappa test. A pre-determined extraction table will be used for data extraction from the included studies for assessment of study quality and evidence synthesis. Extracted

information included: year of publication, the country in which the study took place, the study design (cohort or clinical study), population, duration of follow- up, total number and age range of participants, techniques and samples used for measurement of cytokines, etiology of lung disease, cytokines which were deemed significant, smoking and HIV history (whether on antiretroviral therapy, if/when diagnosed, CD4 cell count, viral load), and any previous hospitalization for lung infection/disease. Extracted information will include: study setting; study population and participant demographics and baseline characteristics; details of the intervention and control conditions; study methodology; recruitment and study completion rates; outcomes and times of measurement; indicators of acceptability to users; suggested mechanisms of intervention action; information for assessment of the risk of bias.

27 Risk of bias (quality) assessment

State whether and how risk of bias will be assessed, how the quality of individual studies will be assessed, and whether and how this will influence the planned synthesis.

Two reviewers will independently assess the risk of bias of the exposure, outcome, and comparisons of each included study using previously established quality assessment scales (the Jadad and the Newcastle-Ottawa Quality Assessment Scales). Cohort studies will be evaluated using three main categories, which include: selection (representativeness of the cohort, ascertainment of exposed individuals, demonstration that the outcome was not present at the start of the study), comparability (cohorts are comparable in design or analysis), and outcome (assessment of outcome and follow-up). Clinical trials will be assessed for: randomization (whether mentioned and appropriate), concealment, blinding (whether mentioned and appropriate), and patient accountability (all patients must be accounted for including withdrawals and dropouts).

28 Strategy for data synthesis

Give the planned general approach to be used, for example whether the data to be used will be aggregate or at the level of individual participants, and whether a quantitative or narrative (descriptive) synthesis is planned. Where appropriate a brief outline of analytic approach should be given.

We will aggregate the information of each study and we will write a narrative synthesis paper. We will not be conducting a meta-analysis.

29 Analysis of subgroups or subsets

Give any planned exploration of subgroups or subsets within the review. ‘None planned’ is a valid response if no subgroup analyses are planned.

None planned

Review general information

30 Type and method of review

Select the type of review and the review method from the drop down list.

Systematic review

Respiratory disorders

31 Language

Select the language(s) in which the review is being written and will be made available, from the drop down list. Use the control key to select more than one language.

English

Will a summary/abstract be made available in English?

Yes

32 Country

Select the country in which the review is being carried out from the drop down list. For multi-national collaborations select all the countries involved. Use the control key to select more than one country.

Canada

33 Other registration details

Give the name of any organisation where the systematic review title or protocol is registered together with any unique identification number assigned. If extracted data will be stored and made available through a repository such as the Systematic Review Data Repository (SRDR), details and a link should be included here.

N/A

34 Reference and/or URL for published protocol

Give the citation for the published protocol, if there is one.
N/A

Give the link to the published protocol, if there is one. This may be to an external site or to a protocol deposited with CRD in pdf format.

I give permission for this file to be made publicly available
Yes

35 Dissemination plans
Give brief details of plans for communicating essential messages from the review to the appropriate audiences.
To publish in an indexed peer-reviewed journal

Do you intend to publish the review on completion?
Yes

36 Keywords
Give words or phrases that best describe the review. (One word per box, create a new box for each term)
lung function

cytokine profile

HIV

pneumonia

37 Details of any existing review of the same topic by the same authors
Give details of earlier versions of the systematic review if an update of an existing review is being registered, including full bibliographic reference if possible.
None

38 Current review status
Review status should be updated when the review is completed and when it is published.
Ongoing

39 Any additional information
Provide any further information the review team consider relevant to the registration of the review.
N/A

40 Details of final report/publication(s)
This field should be left empty until details of the completed review are available.
Give the full citation for the final report or publication of the systematic review.
Give the URL where available.
