# Supplementary material for: Inflammatory mediators and lung abnormalities in HIV: A systematic review
Source: PLoS One. 2019 Dec 12;14(12):e0226347. doi: 10.1371/journal.pone.0226347 (PMC6907827; doi:10.1371/journal.pone.0226347)
Supplement: S2 File — (DOCX) [file pone.0226347.s002.docx]

**Search Strategy:**

1. Lung injury AND HIV

2. Lung injury AND (cytokines OR chemokines OR biomarkers)

3. Lung injury AND (community acquired pneumonia OR pneumonia) AND (cytokines OR chemokines OR biomarkers)

4. Lung injury AND HIV AND (cytokines OR chemokines OR biomarkers)

5. Lung injury AND HIV AND (community acquired pneumonia OR pneumonia) AND (cytokines OR chemokines OR biomarkers)

6. (“Lung function” OR “lung function decline”) AND HIV

7. (“Lung function” OR “lung function decline”) AND (cytokines OR chemokines OR biomarkers)

8. (“Lung function” OR “lung function decline”) AND (community acquired pneumonia OR pneumonia) AND (cytokines OR chemokines OR biomarkers)

9. (“Lung function” OR “lung function decline”) AND HIV AND (cytokines OR chemokines OR biomarkers)

10. (“Lung function” OR “lung function decline”) AND HIV AND (community acquired pneumonia OR pneumonia) AND (cytokines OR chemokines OR biomarkers)

11. Lung inflammation AND HIV

12. Lung inflammation AND (cytokines OR chemokines OR biomarkers)

13. Lung inflammation AND (community acquired pneumonia OR pneumonia) AND (cytokines OR chemokines OR biomarkers)

14. Lung inflammation AND HIV AND (community acquired pneumonia OR pneumonia) AND (cytokines OR chemokines OR biomarkers)

15. Lung inflammation AND HIV AND (cytokines OR chemokines OR biomarkers)

16. Cytokines AND (lung injury OR lung inflammation OR “lung function” OR “lung function decline”)

17. Cytokines AND HIV AND (community acquired pneumonia OR pneumonia)

18. Cytokines AND (lung injury OR lung inflammation OR “lung function” OR “lung function decline”) AND HIV AND (community acquired pneumonia OR pneumonia)

19. Cytokines AND (lung injury OR lung inflammation OR “lung function” OR “lung function decline”) AND (HIV OR community acquired pneumonia OR pneumonia)

20. Cytokines AND (community-acquired pneumonia OR pneumonia)

21. Biomarkers AND (lung injury OR lung inflammation OR “lung function” OR “lung function decline”) AND (HIV OR community acquired pneumonia OR pneumonia)

22. Biomarkers AND (lung injury OR lung inflammation OR “lung function” OR “lung function decline”) AND HIV AND (community acquired pneumonia OR pneumonia)

23. Biomarkers AND HIV AND (community acquired pneumonia OR pneumonia)

24. Biomarkers AND (community-acquired pneumonia OR pneumonia)

Note: For Google Scholar- we restricted the dates to the last four years

**1. Lung injury AND HIV**

**Date**: June 1^st^, 2018

**Database:** Pubmed

**Link**: https://www.ncbi.nlm.nih.gov/pubmed/?cmd=HistorySearch&querykey=13

**Search Details:** "lung injury"[MeSH Major Topic] AND ("hiv"[MeSH Terms] OR "hiv"[All Fields]) AND ((Clinical Study[ptyp] OR Clinical Trial[ptyp] OR Comparative Study[ptyp] OR Observational Study[ptyp]) AND "humans"[MeSH Terms] AND "adult"[MeSH Terms])

**Database:** CENTRAL

**Link**: http://onlinelibrary.wiley.com/cochranelibrary/search

**Search Details:** Search Name: Lung injury AND HIV

Description:

ID Search

#1 Lung injury and HIV

**Database:** Clinical Trials.gov

**Link**:https://clinicaltrials.gov/ct2/show/NCT02058719?term=Lung+injury+AND+HIV&type=Obsr&age=12&hlth=Y&rank=1

**Search Details:** Lung injury AND HIV | Observational Studies | Adult, Senior | Studies that accept healthy volunteers

**Database:** Google Scholar

**Link**: https://scholar-google-com.uml.idm.oclc.org/scholar?as_q=human+adult+18+years+old+HIV&as_epq=lung+injury&as_oq=%22observational+study%22+%22comparative+study%22+%22clinical+study%22+%22clinical+trial%22&as_eq=%22Cystic+fibrosis%22+COPD+cancer+transplant&as_occt=any&as_sauthors=&as_publication=&as_ylo=2014&as_yhi=2018&btnG=&hl=en&as_sdt=1%2C36

**Search Details:** human adult 18 years old HIV "observational study" OR "comparative study" OR "clinical study" OR "clinical trial" "lung injury" -"Cystic fibrosis" -COPD -cancer -transplant

**2. Lung injury AND (cytokines OR chemokines OR biomarkers)**

**Date**: June 1^st^, 2018

**Database:** Pubmed

**Link**: https://www.ncbi.nlm.nih.gov/pubmed/?cmd=HistorySearch&querykey=14

**Search Details:** "lung injury"[MeSH Major Topic] AND (("cytokines"[MeSH Terms] OR "cytokines"[All Fields]) OR ("chemokines"[MeSH Terms] OR "chemokines"[All Fields]) OR ("biomarkers"[MeSH Terms] OR "biomarkers"[All Fields])) AND ((Clinical Study[ptyp] OR Clinical Trial[ptyp] OR Comparative Study[ptyp] OR Observational Study[ptyp]) AND "humans"[MeSH Terms] AND "adult"[MeSH Terms])

**Database:** CENTRAL

**Link**: http://onlinelibrary.wiley.com/cochranelibrary/search

**Search Details:** Search Name: Lung injury AND (cytokines OR chemokines OR biomarkers)

Description:

ID Search

#1 Lung injury and (cytokines or chemokines or biomarkers)

**Database:** Clinical Trials.gov

**Link**:https://clinicaltrials.gov/ct2/results/details?term=Lung+injury+AND+%28cytokines+OR+chemokines+OR+biomarkers%29&type=Obsr&age=1&hlth=Y

**Search Details:** Lung injury AND (cytokines OR chemokines OR biomarkers) | Observational Studies | Adult | Studies that accept healthy volunteers

**Database:** Google Scholar

**Link**: https://scholar-google-com.uml.idm.oclc.org/scholar?as_q=human+adult+18+years+old+cytokine&as_epq=lung+injury&as_oq=%22observational+study%22+%22comparative+study%22+%22clinical+study%22+%22clinical+trial%22&as_eq=COPD+transplant+cancer+lymphoma+%22cystic+fibrosis%22&as_occt=any&as_sauthors=&as_publication=&as_ylo=2014&as_yhi=2018&btnG=&hl=en&as_sdt=1%2C36

**Search Details:** human adult 18 years old cytokine "observational study" OR "comparative study" OR "clinical study" OR "clinical trial" "lung injury" -COPD -transplant -cancer -lymphoma -"cystic fibrosis"

**3. Lung injury AND (community acquired pneumonia OR pneumonia) AND (cytokines OR chemokines OR biomarkers)**

**Date**: June 1^st^, 2018

**Database:** Pubmed

**Link**: https://www.ncbi.nlm.nih.gov/pubmed/?cmd=HistorySearch&querykey=15

**Search Details:** "lung injury"[MeSH Major Topic] AND ((("residence characteristics"[MeSH Terms] OR ("residence"[All Fields] AND "characteristics"[All Fields]) OR "residence characteristics"[All Fields] OR "community"[All Fields]) AND acquired[All Fields] AND ("pneumonia"[MeSH Terms] OR "pneumonia"[All Fields])) OR ("pneumonia"[MeSH Terms] OR "pneumonia"[All Fields])) AND (("cytokines"[MeSH Terms] OR "cytokines"[All Fields]) OR ("chemokines"[MeSH Terms] OR "chemokines"[All Fields]) OR ("biomarkers"[MeSH Terms] OR "biomarkers"[All Fields])) AND ((Clinical Study[ptyp] OR Clinical Trial[ptyp] OR Comparative Study[ptyp] OR Observational Study[ptyp]) AND "humans"[MeSH Terms] AND "adult"[MeSH Terms])

**Database:** CENTRAL

**Link**: http://onlinelibrary.wiley.com/cochranelibrary/search

**Search Details:** Search Name: Lung injury AND (community acquired pneumonia OR pneumonia) AND (cytokines OR chemokines OR biomarkers)

Description:

ID Search

#1 Lung injury and (community acquired pneumonia or pneumonia) and (cytokines or chemokines or biomarkers)

**Database:** Clinical Trials.gov

**Link**:https://clinicaltrials.gov/ct2/results/displayOpt?flds=a&flds=b&flds=i&flds=j&flds=t&flds=g&flds=d&flds=e&submit_fld_opt=on&term=Lung+injury+AND+%28community+acquired+pneumonia+OR+pneumonia%29+AND+%28cytokines+OR+chemokines+OR+biomarkers%29&type=Obsr&age=1&hlth=Y&show_flds=Y

**Search Details:** Lung injury AND (community acquired pneumonia OR pneumonia) AND (cytokines OR chemokines OR biomarkers) | Observational Studies | Adult | Studies that accept healthy volunteers

**Database:** Google Scholar

**Link**: https://scholar-google-com.uml.idm.oclc.org/scholar?as_q=human+adult+18+years+old+cytokine+pneumonia&as_epq=lung+injury&as_oq=%22observational+study%22+%22comparative+study%22+%22clinical+study%22+%22clinical+trial%22&as_eq=%22Cystic+fibrosis%22+COPD+cancer+transplant&as_occt=any&as_sauthors=&as_publication=&as_ylo=2014&as_yhi=2018&btnG=&hl=en&as_sdt=1%2C36

**Search Details**: human adult 18 years old cytokine pneumonia "observational study" OR "comparative study" OR "clinical study" OR "clinical trial" "lung injury" -"Cystic fibrosis" -COPD -cancer –transplant

**4. Lung injury AND HIV AND (cytokines OR chemokines OR biomarkers)**

**Date**: June 1^st^, 2018

**Database:** Pubmed

**Link**: https://www.ncbi.nlm.nih.gov/pubmed/?cmd=HistorySearch&querykey=16

**Search Details:** "lung injury"[MeSH Major Topic] AND ("hiv"[MeSH Terms] OR "hiv"[All Fields]) AND (("cytokines"[MeSH Terms] OR "cytokines"[All Fields]) OR ("chemokines"[MeSH Terms] OR "chemokines"[All Fields]) OR ("biomarkers"[MeSH Terms] OR "biomarkers"[All Fields])) AND ((Clinical Study[ptyp] OR Clinical Trial[ptyp] OR Comparative Study[ptyp] OR Observational Study[ptyp]) AND "humans"[MeSH Terms] AND "adult"[MeSH Terms])

**Database:** CENTRAL

**Link**: http://onlinelibrary.wiley.com/cochranelibrary/search

**Search Details:** Search Name: Lung injury (MeSH) AND HIV AND (cytokines OR chemokines OR biomarkers)

Description:

ID Search

#1 Lung injury and HIV and (cytokines or chemokines or biomarkers)

**Database:** Clinical Trials.gov

**Link**:https://clinicaltrials.gov/ct2/results?term=Lung+injury+AND+HIV+AND+%28cytokines+OR+chemokines+OR+biomarkers%29&type=Obsr&rslt=&recr=&age_v=&age=1&gndr=&hlth=Y&cond=&intr=&titles=&outc=&spons=&lead=&id=&state1=&cntry1=&state2=&cntry2=&state3=&cntry3=&locn=&rcv_s=&rcv_e=&lup_s=&lup_e=

**Search Details:** Lung injury AND HIV AND (cytokines OR chemokines OR biomarkers) | Observational Studies | Adult | Studies that accept healthy volunteers

**Database:** Google Scholar

**Link**: https://scholar-google-com.uml.idm.oclc.org/scholar?as_q=HIV+adult+18+years+old+cytokine+human&as_epq=lung+injury&as_oq=%22observational+study%22+%22comparative+study%22+%22clinical+study%22+%22clinical+trial%22&as_eq=%22Cystic+fibrosis%22+COPD+cancer+transplant&as_occt=any&as_sauthors=&as_publication=&as_ylo=2014&as_yhi=2018&btnG=&hl=en&as_sdt=1%2C36

**Search Details:** HIV adult 18 years old cytokine human "observational study" OR "comparative study" OR "clinical study" OR "clinical trial" "lung injury" -"Cystic fibrosis" -COPD -cancer -transplant

**5. Lung injury AND HIV AND (community acquired pneumonia OR pneumonia) AND (cytokines OR chemokines OR biomarkers)**

**Date**: June 1^st^, 2018

**Database:** Pubmed

**Link**: https://www.ncbi.nlm.nih.gov/pubmed/?cmd=HistorySearch&querykey=18

**Search Details:** "lung injury"[MeSH Major Topic] AND ("hiv"[MeSH Terms] OR "hiv"[All Fields]) AND ((("residence characteristics"[MeSH Terms] OR ("residence"[All Fields] AND "characteristics"[All Fields]) OR "residence characteristics"[All Fields] OR "community"[All Fields]) AND acquired[All Fields] AND ("pneumonia"[MeSH Terms] OR "pneumonia"[All Fields])) OR ("pneumonia"[MeSH Terms] OR "pneumonia"[All Fields])) AND (("cytokines"[MeSH Terms] OR "cytokines"[All Fields]) OR ("chemokines"[MeSH Terms] OR "chemokines"[All Fields]) OR ("biomarkers"[MeSH Terms] OR "biomarkers"[All Fields])) AND ((Clinical Study[ptyp] OR Clinical Trial[ptyp] OR Comparative Study[ptyp] OR Observational Study[ptyp]) AND "humans"[MeSH Terms] AND "adult"[MeSH Terms])

**Database:** CENTRAL

**Link**: http://onlinelibrary.wiley.com/cochranelibrary/search

**Search Details:** Lung injury (MeSH) and HIV and (community acquired pneumonia or pneumonia) and (cytokines or chemokines or biomarkers)

Description:

ID Search

#1 Lung injury and HIV and (community acquired pneumonia or pneumonia) and (cytokines or chemokines or biomarkers)

**Database:** Clinical Trials.gov

**Link**:https://clinicaltrials.gov/ct2/results?term=Lung+injury+AND+HIV+AND+%28community+acquired+pneumonia+OR+pneumonia%29+AND+%28cytokines+OR+chemokines+OR+biomarkers%29&type=Obsr&rslt=&recr=&age_v=&age=1&gndr=&hlth=Y&cond=&intr=&titles=&outc=&spons=&lead=&id=&state1=&cntry1=&state2=&cntry2=&state3=&cntry3=&locn=&rcv_s=&rcv_e=&lup_s=&lup_e=

**Search Details:** Lung injury AND HIV AND (community acquired pneumonia OR pneumonia) AND (cytokines OR chemokines OR biomarkers) | Observational Studies | Adult | Studies that accept healthy volunteers

**Database:** Google Scholar

**Link**: https://scholar-google-com.uml.idm.oclc.org/scholar?as_q=HIV+pneumonia+human+adult+18+years+old+cytokine+&as_epq=lung+injury&as_oq=%22observational+study%22+%22comparative+study%22+%22clinical+study%22+%22clinical+trial%22&as_eq=%22Cystic+fibrosis%22+COPD+cancer+transplant&as_occt=any&as_sauthors=&as_publication=&as_ylo=2014&as_yhi=2018&btnG=&hl=en&as_sdt=1%2C36

**Search Details:** HIV pneumonia human adult 18 years old cytokine "observational study" OR "comparative study" OR "clinical study" OR "clinical trial" "lung injury" -"Cystic fibrosis" -COPD -cancer -transplant

**6. (“Lung function” OR “lung function decline”) AND HIV**

**Date**: June 1^st^, 2018

**Database:** Pubmed

**Link**: https://www.ncbi.nlm.nih.gov/pubmed/?cmd=HistorySearch&querykey=20

**Search Details:** ("Lung function"[All Fields] OR "lung function decline"[All Fields]) AND ("hiv"[MeSH Terms] OR "hiv"[All Fields]) AND ((Clinical Study[ptyp] OR Clinical Trial[ptyp] OR Comparative Study[ptyp] OR Observational Study[ptyp]) AND "humans"[MeSH Terms] AND "adult"[MeSH Terms])

**Database:** CENTRAL

**Link**: http://onlinelibrary.wiley.com/cochranelibrary/search

**Search Details:** (“Lung function” OR “lung function decline”) AND HIV

Description:

ID Search

#1 ("Lung function" or "lung function decline") and HIV

**Database:** Clinical Trials.gov

**Link**:https://clinicaltrials.gov/ct2/results?term=%28“Lung+function”+OR+“lung+function+decline”%29+AND+HIV&type=Obsr&age=1&hlth=Y

**Search Details:** (“Lung function” OR “lung function decline”) AND HIV | Observational Studies | Adult | Studies that accept healthy volunteers

**Database:** Google Scholar

**Link**: https://scholar-google-com.uml.idm.oclc.org/scholar?as_q=HIV+human+adult+18+years+old&as_epq=lung+function&as_oq=%22observational+study%22+%22comparative+study%22+%22clinical+study%22+%22clinical+trial%22&as_eq=%22Cystic+fibrosis%22+COPD+cancer+transplant&as_occt=any&as_sauthors=&as_publication=&as_ylo=2014&as_yhi=2018&btnG=&hl=en&as_sdt=1%2C36

**Search Details:** HIV human adult 18 years old "observational study" OR "comparative study" OR "clinical study" OR "clinical trial" "lung function" -"Cystic fibrosis" -COPD -cancer -transplant

**7. (“Lung function” OR “lung function decline”) AND (cytokines OR chemokines OR biomarkers)**

**Date**: June 1^st^, 2018

**Database:** Pubmed

**Link**: https://www.ncbi.nlm.nih.gov/pubmed/?cmd=HistorySearch&querykey=25

**Search Details:** ("Lung function"[All Fields] OR "lung function decline"[All Fields]) AND (("cytokines"[MeSH Terms] OR "cytokines"[All Fields]) OR ("chemokines"[MeSH Terms] OR "chemokines"[All Fields]) OR ("biomarkers"[MeSH Terms] OR "biomarkers"[All Fields])) AND ((Clinical Study[ptyp] OR Clinical Trial[ptyp] OR Comparative Study[ptyp] OR Observational Study[ptyp]) AND "humans"[MeSH Terms] AND "adult"[MeSH Terms])

**Database:** CENTRAL

**Link**: http://onlinelibrary.wiley.com/cochranelibrary/search

**Search Details:** (“Lung function” OR “lung function decline”) AND (cytokines OR chemokines OR biomarkers)

Description:

ID Search

#1 ("Lung function" or "lung function decline") and (cytokines or chemokines or biomarkers)

**Database:** Clinical Trials.gov

**Link**:https://clinicaltrials.gov/ct2/results?term=%28“Lung+function”+OR+“lung+function+decline”%29+AND+%28cytokines+OR+chemokines+OR+biomarkers%29&type=Obsr&age=1&hlth=Y

**Search Details:** (“Lung function” OR “lung function decline”) AND (cytokines OR chemokines OR biomarkers) | Observational Studies | Adult | Studies that accept healthy volunteers

**Database:** Google Scholar

**Link**: https://scholar-google-com.uml.idm.oclc.org/scholar?as_q=human+adult+18+years+old+cytokine&as_epq=lung+function&as_oq=%22observational+study%22+%22comparative+study%22+%22clinical+study%22+%22clinical+trial%22&as_eq=%22Cystic+fibrosis%22+COPD+cancer+transplant&as_occt=any&as_sauthors=&as_publication=&as_ylo=2014&as_yhi=2018&btnG=&hl=en&as_sdt=1%2C36

**Search Details:** Human adult 18 years old cytokine "observational study" OR "comparative study" OR "clinical study" OR "clinical trial" "lung function" -"Cystic fibrosis" -COPD -cancer -transplant

**8. (“Lung function” OR “lung function decline”) AND (community acquired pneumonia OR pneumonia) AND (cytokines OR chemokines OR biomarkers)**

**Date**: June 1^st^, 2018

**Database:** Pubmed

**Link**: https://www.ncbi.nlm.nih.gov/pubmed/?cmd=HistorySearch&querykey=33

**Search Details:** ("Lung function"[All Fields] OR "lung function decline"[All Fields]) AND ((("residence characteristics"[MeSH Terms] OR ("residence"[All Fields] AND "characteristics"[All

Fields]) OR "residence characteristics"[All Fields] OR "community"[All Fields]) AND acquired[All Fields] AND ("pneumonia"[MeSH Terms] OR "pneumonia"[All Fields])) OR ("pneumonia"[MeSH Terms] OR "pneumonia"[All Fields])) AND (("cytokines"[MeSH Terms] OR "cytokines"[All Fields]) OR ("chemokines"[MeSH Terms] OR "chemokines"[All Fields]) OR ("biomarkers"[MeSH Terms] OR "biomarkers"[All Fields])) AND ((Clinical Study[ptyp] OR Clinical Trial[ptyp] OR Comparative Study[ptyp] OR Observational Study[ptyp]) AND "humans"[MeSH Terms] AND "adult"[MeSH Terms])

**Database:** CENTRAL

**Link**: http://onlinelibrary.wiley.com/cochranelibrary/search

**Search Details:** ("Lung function" OR "lung function decline") AND (community acquired pneumonia OR pneumonia) AND (cytokines OR chemokines OR biomarkers)

Description:

ID Search

#1 ("Lung function" or "lung function decline") and (community acquired pneumonia or pneumonia) and (cytokines or chemokines or biomarkers)

**Database:** Clinical Trials.gov

**Link**:https://clinicaltrials.gov/ct2/results?term=%28“Lung+function”+OR+“lung+function+decline”%29+AND+%28community+acquired+pneumonia+OR+pneumonia%29+AND+%28cytokines+OR+chemokines+OR+biomarkers%29&type=Obsr&rslt=&recr=&age_v=&age=1&gndr=&hlth=Y&cond=&intr=&titles=&outc=&spons=&lead=&id=&state1=&cntry1=&state2=&cntry2=&state3=&cntry3=&locn=&rcv_s=&rcv_e=&lup_s=&lup_e=

**Search Details:** (“Lung function” OR “lung function decline”) AND (community acquired pneumonia OR pneumonia) AND (cytokines OR chemokines OR biomarkers) | Observational Studies | Adult | Studies that accept healthy volunteers

**Database:** Google Scholar

**Link**: https://scholar-google-com.uml.idm.oclc.org/scholar?as_q=human+adult+18+years+old+cytokine+pneumonia&as_epq=lung+function&as_oq=%22observational+study%22+%22comparative+study%22+%22clinical+study%22+%22clinical+trial%22&as_eq=%22Cystic+fibrosis%22+COPD+cancer+transplant+neonate+child&as_occt=any&as_sauthors=&as_publication=&as_ylo=2014&as_yhi=2018&btnG=&hl=en&as_sdt=1%2C36

**Search Details:** human adult 18 years old cytokine pneumonia "observational study" OR "comparative study" OR "clinical study" OR "clinical trial" "lung function" -"Cystic fibrosis" -COPD -cancer -transplant -neonate -child

**9. (“Lung function” OR “lung function decline”) AND HIV AND (cytokines OR chemokines OR biomarkers)**

**Date**: June 1^st^, 2018

**Database:** Pubmed

**Link**: https://www.ncbi.nlm.nih.gov/pubmed/?cmd=HistorySearch&querykey=34

**Search Details:** ("Lung function"[All Fields] OR "lung function decline"[All Fields]) AND ("hiv"[MeSH Terms] OR "hiv"[All Fields]) AND (("cytokines"[MeSH Terms] OR "cytokines"[All Fields]) OR ("chemokines"[MeSH Terms] OR "chemokines"[All Fields]) OR ("biomarkers"[MeSH Terms] OR "biomarkers"[All Fields])) AND ((Clinical Study[ptyp] OR Clinical Trial[ptyp] OR Comparative Study[ptyp] OR Observational Study[ptyp]) AND "humans"[MeSH Terms] AND "adult"[MeSH Terms])

**Database:** CENTRAL

**Link**: http://onlinelibrary.wiley.com/cochranelibrary/search

**Search Details:** (“Lung function” OR “lung function decline”) AND HIV AND (cytokines OR chemokines OR biomarkers)

Description:

ID Search

#1 ("Lung function" or "lung function decline") and HIV and (cytokines or chemokines or biomarkers)

**Database:** Clinical Trials.gov

**Link**:https://clinicaltrials.gov/ct2/results?term=%28“Lung+function”+OR+“lung+function+decline”%29+AND+HIV+AND+%28cytokines+OR+chemokines+OR+biomarkers%29&type=Obsr&rslt=&recr=&age_v=&age=1&gndr=&hlth=Y&cond=&intr=&titles=&outc=&spons=&lead=&id=&state1=&cntry1=&state2=&cntry2=&state3=&cntry3=&locn=&rcv_s=&rcv_e=&lup_s=&lup_e=

**Search Details:** (“Lung function” OR “lung function decline”) AND HIV AND (cytokines OR chemokines OR biomarkers) | Observational Studies | Adult | Studies that accept healthy volunteers

**Database:** Google Scholar

**Link**: https://scholar-google-com.uml.idm.oclc.org/scholar?as_q=HIV+human+adult+18+years+old+cytokine&as_epq=lung+function&as_oq=%22observational+study%22+%22comparative+study%22+%22clinical+study%22+%22clinical+trial%22&as_eq=%22Cystic+fibrosis%22+COPD+cancer+transplant+neonate+child&as_occt=any&as_sauthors=&as_publication=&as_ylo=2014&as_yhi=2018&hl=en&as_sdt=1%2C36

**Search Details:** HIV human adult 18 years old cytokine "observational study" OR "comparative study" OR "clinical study" OR "clinical trial" "lung function" -"Cystic fibrosis" -COPD -cancer -transplant -neonate -child

**10. (“Lung function” OR “lung function decline”) AND HIV AND (community acquired pneumonia OR pneumonia) AND (cytokines OR chemokines OR biomarkers)**

**Date**: June 1^st^, 2018

**Database:** Pubmed

**Link**: https://www.ncbi.nlm.nih.gov/pubmed/?cmd=HistorySearch&querykey=36

**Search Details:** ("Lung function"[All Fields] OR "lung function decline"[All Fields]) AND ("hiv"[MeSH Terms] OR "hiv"[All Fields]) AND ((("residence characteristics"[MeSH Terms] OR ("residence"[All Fields] AND "characteristics"[All Fields]) OR "residence characteristics"[All

Fields] OR "community"[All Fields]) AND acquired[All Fields] AND ("pneumonia"[MeSH Terms] OR "pneumonia"[All Fields])) OR ("pneumonia"[MeSH Terms] OR "pneumonia"[All Fields])) AND (("cytokines"[MeSH Terms] OR "cytokines"[All Fields]) OR ("chemokines"[MeSH Terms] OR "chemokines"[All Fields]) OR ("biomarkers"[MeSH Terms] OR "biomarkers"[All Fields])) AND ((Clinical Study[ptyp] OR Clinical Trial[ptyp] OR Comparative Study[ptyp] OR Observational Study[ptyp]) AND "humans"[MeSH Terms] AND "adult"[MeSH Terms])

**Database:** CENTRAL

**Link**: http://onlinelibrary.wiley.com/cochranelibrary/search

**Search Details:** ("Lung function" OR "lung function decline") AND HIV AND (community acquired pneumonia OR pneumonia) AND (cytokines OR chemokines OR biomarkers)

Description:

ID Search

#1 ("Lung function" or "lung function decline") and HIV and (community acquired pneumonia or pneumonia) and (cytokines or chemokines or biomarkers)

**Database:** Clinical Trials.gov

**Link**:https://clinicaltrials.gov/ct2/results?term=%28Lung+function+OR+lung+function+decline%29+AND+HIV+AND+%28community+acquired+pneumonia+OR+pneumonia%29+AND+%28cytokines+OR+chemokines+OR+biomarkers%29&type=Obsr&rslt=&recr=&age_v=&age=1&gndr=&hlth=Y&cond=&intr=&titles=&outc=&spons=&lead=&id=&state1=&cntry1=&state2=&cntry2=&state3=&cntry3=&locn=&rcv_s=&rcv_e=&lup_s=&lup_e=

**Search Details:** (Lung function OR lung function decline) AND HIV AND (community acquired pneumonia OR pneumonia) AND (cytokines OR chemokines OR biomarkers) | Observational Studies | Adult | Studies that accept healthy volunteers

**Database:** Google Scholar

**Link**: https://scholar-google-com.uml.idm.oclc.org/scholar?as_q=human+adult+18+years+old+cytokine+HIV+pneumonia&as_epq=lung+function&as_oq=%22observational+study%22+%22comparative+study%22+%22clinical+study%22+%22clinical+trial%22&as_eq=%22Cystic+fibrosis%22+COPD+cancer+transplant+neonate+child&as_occt=any&as_sauthors=&as_publication=&as_ylo=2014&as_yhi=2018&btnG=&hl=en&as_sdt=1%2C36

**Search Details:** human adult 18 years old cytokine HIV pneumonia "observational study" OR "comparative study" OR "clinical study" OR "clinical trial" "lung function" -"Cystic fibrosis" -COPD -cancer -transplant -neonate -child

**11. Lung inflammation AND HIV**

**Date**: June 1^st^, 2018

**Database:** Pubmed

**Link**: https://www.ncbi.nlm.nih.gov/pubmed/?cmd=HistorySearch&querykey=28

**Search Details:** "pneumonia"[MeSH Major Topic] AND ("hiv"[MeSH Terms] OR "hiv"[All Fields]) AND ((Clinical Study[ptyp] OR Clinical Trial[ptyp] OR Comparative Study[ptyp] OR Observational Study[ptyp]) AND "humans"[MeSH Terms] AND "adult"[MeSH Terms])

**Database:** CENTRAL

**Link**: http://onlinelibrary.wiley.com/cochranelibrary/search

**Search Details:** Lung inflammation and HIV

Description:

ID Search

#1 Lung inflammation and HIV

**Database:** Clinical Trials.gov

**Link**:https://clinicaltrials.gov/ct2/results?term=Lung+inflammation+AND+HIV&type=Obsr&rslt=&recr=&age_v=&age=1&gndr=&hlth=Y&cond=&intr=&titles=&outc=&spons=&lead=&id=&state1=&cntry1=&state2=&cntry2=&state3=&cntry3=&locn=&rcv_s=&rcv_e=&lup_s=&lup_e=

**Search Details:** Lung inflammation AND HIV | Observational Studies | Adult | Studies that accept healthy volunteers

**Database:** Google Scholar

**Link**: https://scholar-google-com.uml.idm.oclc.org/scholar?as_q=human+adult+18+years+old+HIV&as_epq=lung+inflammation&as_oq=%22observational+study%22+%22comparative+study%22+%22clinical+study%22+%22clinical+trial%22&as_eq=%22Cystic+fibrosis%22+COPD+cancer+transplant+neonate+child&as_occt=any&as_sauthors=&as_publication=&as_ylo=2014&as_yhi=2018&hl=en&as_sdt=1%2C36

**Search Details:** Human adult 18 years old HIV "observational study" OR "comparative study" OR "clinical study" OR "clinical trial" "lung inflammation" -"Cystic fibrosis" -COPD -cancer -transplant -neonate –child

**12. Lung inflammation AND (cytokines OR chemokines OR biomarkers)**

**Date**: June 1^st^, 2018

**Database:** Pubmed

**Link**: https://www.ncbi.nlm.nih.gov/pubmed/?cmd=HistorySearch&querykey=29

**Search Details:** "pneumonia"[MeSH Major Topic] AND (("cytokines"[MeSH Terms] OR "cytokines"[All Fields]) OR ("chemokines"[MeSH Terms] OR "chemokines"[All Fields]) OR ("biomarkers"[MeSH Terms] OR "biomarkers"[All Fields])) AND ((Clinical Study[ptyp] OR Clinical Trial[ptyp] OR Comparative Study[ptyp] OR Observational Study[ptyp]) AND "humans"[MeSH Terms] AND "adult"[MeSH Terms])

**Database:** CENTRAL

**Link**: http://onlinelibrary.wiley.com/cochranelibrary/search

**Search Details:** Lung inflammation AND (cytokines OR chemokines OR biomarkers)

Description:

ID Search

#1 Lung inflammation and (cytokines or chemokines or biomarkers)

**Database:** Clinical Trials.gov

**Link**:https://clinicaltrials.gov/ct2/results?term=Lung+inflammation+AND+%28cytokines+OR+chemokines+OR+biomarkers%29&type=Obsr&rslt=&recr=&age_v=&age=1&gndr=&hlth=Y&cond=&intr=&titles=&outc=&spons=&lead=&id=&state1=&cntry1=&state2=&cntry2=&state3=&cntry3=&locn=&rcv_s=&rcv_e=&lup_s=&lup_e=

**Search Details:** Lung inflammation AND (cytokines OR chemokines OR biomarkers) | Observational Studies | Adult | Studies that accept healthy volunteers

**Database:** Google Scholar

**Link**:https://scholar.google.ca/scholar?as_q=human+adult+18+years+old+cytokine&as_epq=lung+inflammation&as_oq=%22observational+study%22+%22comparative+study%22+%22clinical+study%22+%22clinical+trial%22&as_eq=%22Cystic+fibrosis%22+COPD+cancer+transplant+neonate+child&as_occt=any&as_sauthors=&as_publication=&as_ylo=&as_yhi=&btnG=&hl=en&as_sdt=0%2C5

**Search Details:** human adult 18 years old cytokine "observational study" OR "comparative study" OR "clinical study" OR "clinical trial" "lung inflammation" -"Cystic fibrosis" -COPD -cancer -transplant -neonate –child

**13. Lung inflammation AND (community acquired pneumonia OR pneumonia) AND (cytokines OR chemokines OR biomarkers)**

**Date**: June 1^st^, 2018

**Database:** Pubmed

**Link**: https://www.ncbi.nlm.nih.gov/pubmed/?cmd=HistorySearch&querykey=30

**Search Details:** "pneumonia"[MeSH Major Topic] AND ((("residence characteristics"[MeSH Terms] OR ("residence"[All Fields] AND "characteristics"[All Fields]) OR "residence characteristics"[All Fields] OR "community"[All Fields]) AND acquired[All Fields] AND ("pneumonia"[MeSH Terms] OR "pneumonia"[All Fields])) OR ("pneumonia"[MeSH Terms] OR "pneumonia"[All Fields])) AND (("cytokines"[MeSH Terms] OR "cytokines"[All Fields]) OR ("chemokines"[MeSH Terms] OR "chemokines"[All Fields]) OR ("biomarkers"[MeSH Terms] OR "biomarkers"[All Fields])) AND ((Clinical Study[ptyp] OR Clinical Trial[ptyp] OR Comparative Study[ptyp] OR Observational Study[ptyp]) AND "humans"[MeSH Terms] AND "adult"[MeSH Terms])

**Database:** CENTRAL

**Link**: http://onlinelibrary.wiley.com/cochranelibrary/search

**Search Details:** Lung inflammation and (community acquired pneumonia or pneumonia) and (cytokines or chemokines or biomarkers)

Description:

ID Search

#1 Lung inflammation and (community acquired pneumonia or pneumonia) and (cytokines or chemokines or biomarkers)

**Database:** Clinical Trials.gov

**Link**:https://clinicaltrials.gov/ct2/results?term=Lung+inflammation+AND+%28community+acquired+pneumonia+OR+pneumonia%29+AND+%28cytokines+OR+chemokines+OR+biomarkers%2

9&type=Obsr&rslt=&recr=&age_v=&age=1&gndr=&hlth=Y&cond=&intr=&titles=&outc=&spons=&lead=&id=&state1=&cntry1=&state2=&cntry2=&state3=&cntry3=&locn=&rcv_s=&rcv_e=&lup_s=&lup_e=

**Search Details:** Lung inflammation AND (community acquired pneumonia OR pneumonia) AND (cytokines OR chemokines OR biomarkers) | Observational Studies | Adult | Studies that accept healthy volunteers

**Database:** Google Scholar

**Link**:https://scholar.google.ca/scholar?as_q=Human+adult+cytokine+18+years+old+pneumonia&as_epq=lung+inflammation&as_oq=%22observational+study%22+%22comparative+study%22+%22clinical+study%22+%22clinical+trial%22&as_eq=%22Cystic+fibrosis%22+COPD+cancer+transplant+neonate+child&as_occt=any&as_sauthors=&as_publication=&as_ylo=&as_yhi=&btnG=&hl=en&as_sdt=0%2C5

**Search Details:** Human adult cytokine 18 years old pneumonia "observational study" OR "comparative study" OR "clinical study" OR "clinical trial" "lung inflammation" -"Cystic fibrosis" -COPD -cancer -transplant -neonate –child

**14. Lung inflammation AND HIV AND (community acquired pneumonia OR pneumonia) AND (cytokines OR chemokines OR biomarkers)**

**Date**: June 1^st^, 2018

**Database:** Pubmed

**Link**: https://www.ncbi.nlm.nih.gov/pubmed/?cmd=HistorySearch&querykey=31

**Search Details:** "pneumonia"[MeSH Major Topic] AND ("hiv"[MeSH Terms] OR "hiv"[All Fields]) AND ((("residence characteristics"[MeSH Terms] OR ("residence"[All Fields] AND "characteristics"[All Fields]) OR "residence characteristics"[All Fields] OR "community"[All Fields]) AND acquired[All Fields] AND ("pneumonia"[MeSH Terms] OR "pneumonia"[All Fields])) OR ("pneumonia"[MeSH Terms] OR "pneumonia"[All Fields])) AND (("cytokines"[MeSH Terms] OR "cytokines"[All Fields]) OR ("chemokines"[MeSH Terms] OR "chemokines"[All Fields]) OR ("biomarkers"[MeSH Terms] OR "biomarkers"[All Fields])) AND ((Clinical Study[ptyp] OR Clinical Trial[ptyp] OR Comparative Study[ptyp] OR Observational Study[ptyp]) AND "humans"[MeSH Terms] AND "adult"[MeSH Terms])

**Database:** CENTRAL

**Link**: http://onlinelibrary.wiley.com/cochranelibrary/search

**Search Details:** Lung inflammation AND HIV AND (community acquired pneumonia OR pneumonia) AND (cytokines OR chemokines OR biomarkers)

Description:

ID Search

#1 Lung inflammation and HIV and (community acquired pneumonia or pneumonia) and (cytokines or chemokines or biomarkers)

**Database:** Clinical Trials.gov

**Link**:https://clinicaltrials.gov/ct2/results?term=Lung+inflammation+AND+HIV+AND+%28community+acquired+pneumonia+OR+pneumonia%29+AND+%28cytokines+OR+chemokines+OR+bio

markers%29&type=Obsr&rslt=&recr=&age_v=&age=1&gndr=&hlth=Y&cond=&intr=&titles=&outc=&spons=&lead=&id=&state1=&cntry1=&state2=&cntry2=&state3=&cntry3=&locn=&rcv_s=&rcv_e=&lup_s=&lup_e=

**Search Details:** Lung inflammation AND HIV AND (community acquired pneumonia OR pneumonia) AND (cytokines OR chemokines OR biomarkers) | Observational Studies | Adult | Studies that accept healthy volunteers

**Database:** Google Scholar

**Link**:https://scholar.google.ca/scholar?as_q=Human+adult+cytokine+18+years+old+HIV+pneumonia&as_epq=lung+inflammation&as_oq=%22observational+study%22+%22comparative+study%22+%22clinical+study%22+%22clinical+trial%22&as_eq=%22Cystic+fibrosis%22+COPD+cancer+transplant+neonate+child&as_occt=any&as_sauthors=&as_publication=&as_ylo=&as_yhi=&btnG=&hl=en&as_sdt=0%2C5

**Search Details:** Human adult cytokine 18 years old HIV pneumonia "observational study" OR "comparative study" OR "clinical study" OR "clinical trial" "lung inflammation" -"Cystic fibrosis" -COPD -cancer -transplant -neonate -child

**15. Lung inflammation AND HIV AND (cytokines OR chemokines OR biomarkers)**

**Date**: June 1^st^, 2018

**Link**: https://www.ncbi.nlm.nih.gov/pubmed/?cmd=HistorySearch&querykey=32

**Search Details:** "pneumonia"[MeSH Major Topic] AND ("hiv"[MeSH Terms] OR "hiv"[All Fields]) AND (("cytokines"[MeSH Terms] OR "cytokines"[All Fields]) OR ("chemokines"[MeSH Terms] OR "chemokines"[All Fields]) OR ("biomarkers"[MeSH Terms] OR "biomarkers"[All Fields])) AND ((Clinical Study[ptyp] OR Clinical Trial[ptyp] OR Comparative Study[ptyp] OR Observational Study[ptyp]) AND "humans"[MeSH Terms] AND "adult"[MeSH Terms])

**Database:** CENTRAL

**Link**: http://onlinelibrary.wiley.com/cochranelibrary/search

**Search Details:** Lung inflammation AND HIV AND (cytokines OR chemokines OR biomarkers)

Description:

ID Search

#1 Lung inflammation and HIV and (cytokines or chemokines or biomarkers)

**Database:** Clinical Trials.gov

**Link**:https://clinicaltrials.gov/ct2/results?term=Lung+inflammation+AND+HIV+AND+%28cytokines+OR+chemokines+OR+biomarkers%29&type=Obsr&rslt=&recr=&age_v=&age=1&gndr=&hlth=Y&cond=&intr=&titles=&outc=&spons=&lead=&id=&state1=&cntry1=&state2=&cntry2=&state3=&cntry3=&locn=&rcv_s=&rcv_e=&lup_s=&lup_e=

**Search Details:** Lung inflammation AND HIV AND (cytokines OR chemokines OR biomarkers) | Observational Studies | Adult | Studies that accept healthy volunteers

**Database:** Google Scholar

**Link**:https://scholar.google.ca/scholar?as_q=Human+adult+cytokine+HIV+18+years+old&as_epq=lung+inflammation&as_oq=%22observational+study%22+%22comparative+study%22+%22cli

nical+study%22+%22clinical+trial%22&as_eq=%22Cystic+fibrosis%22+COPD+cancer+transplant+neonate+child&as_occt=any&as_sauthors=&as_publication=&as_ylo=&as_yhi=&btnG=&hl=en&as_sdt=0%2C5

**Search Details:** Human adult cytokine HIV 18 years old "observational study" OR "comparative study" OR "clinical study" OR "clinical trial" "lung inflammation" -"Cystic fibrosis" -COPD -cancer -transplant -neonate -child

**16. Cytokines AND (lung injury OR lung inflammation OR “lung function” OR “lung function decline”)**

**Date**: June 1^st^, 2018

**Database:** Pubmed

**Link**: https://www.ncbi.nlm.nih.gov/pubmed/?cmd=HistorySearch&querykey=42

**Search Details:** "cytokines"[MeSH Major Topic] AND (("lung injury"[MeSH Terms] OR ("lung"[All Fields] AND "injury"[All Fields]) OR "lung injury"[All Fields]) OR ("pneumonia"[MeSH Terms] OR "pneumonia"[All Fields] OR ("lung"[All Fields] AND "inflammation"[All Fields]) OR "lung inflammation"[All Fields]) OR "lung function"[All Fields] OR "lung function decline"[All Fields]) AND ((Clinical Study[ptyp] OR Clinical Trial[ptyp] OR Comparative Study[ptyp] OR Observational Study[ptyp]) AND "humans"[MeSH Terms] AND "adult"[MeSH Terms])

**Database:** CENTRAL

**Link**: http://onlinelibrary.wiley.com/cochranelibrary/search

**Search Details:** Cytokines AND (lung injury OR lung inflammation OR "lung function" OR "lung function decline")

Description:

ID Search

#1 Cytokines and (lung injury or lung inflammation or "lung function" or "lung function decline")

**Database:** Clinical Trials.gov

**Link**:https://clinicaltrials.gov/ct2/results?term=Cytokines++AND+%28lung+injury+OR+lung+inflammation+OR+lung+function+OR+lung+function+decline%29&type=Obsr&rslt=&recr=&age_v=&age=1&gndr=&hlth=Y&cond=&intr=&titles=&outc=&spons=&lead=&id=&state1=&cntry1=&state2=&cntry2=&state3=&cntry3=&locn=&rcv_s=&rcv_e=&lup_s=&lup_e=

**Search Details:** Cytokines AND (lung injury OR lung inflammation OR lung function OR lung function decline) | Observational Studies | Adult | Studies that accept healthy volunteers

**Database:** Google Scholar

**Link**:https://scholar.google.ca/scholar?as_q=Human+adult+cytokine+18+years+old&as_epq=lung+inflammation&as_oq=%22observational+study%22+%22comparative+study%22+%22clinical+study%22+%22clinical+trial%22&as_eq=%22Cystic+fibrosis%22+COPD+cancer+transplant+neonate+child&as_occt=any&as_sauthors=&as_publication=&as_ylo=&as_yhi=&btnG=&hl=en&as_sdt=0%2C5

**Search Details:** Human adult cytokine 18 years old "observational study" OR "comparative study" OR "clinical study" OR "clinical trial" "lung inflammation" -"Cystic fibrosis" -COPD -cancer -transplant -neonate -child

**17. Cytokines AND HIV AND (community acquired pneumonia OR pneumonia)**

**Date**: June 1^st^, 2018

**Database:** PubMed

**Link**: https://www.ncbi.nlm.nih.gov/pubmed

**Search Details:** "cytokines"[MeSH Major Topic] AND ("hiv"[MeSH Terms] OR "hiv"[All Fields]) AND ((("residence characteristics"[MeSH Terms] OR ("residence"[All Fields] AND "characteristics"[All Fields]) OR "residence characteristics"[All Fields] OR "community"[All Fields]) AND acquired[All Fields] AND ("pneumonia"[MeSH Terms] OR "pneumonia"[All Fields])) OR ("pneumonia"[MeSH Terms] OR "pneumonia"[All Fields])) AND ((Clinical Study[ptyp] OR Clinical Trial[ptyp] OR Comparative Study[ptyp] OR Observational Study[ptyp]) AND "humans"[MeSH Terms] AND "adult"[MeSH Terms])

**Database:** CENTRAL

**Link**: http://onlinelibrary.wiley.com/cochranelibrary/search

**Search Details:** Cytokines AND HIV AND (community acquired pneumonia OR pneumonia)

Description:

ID Search

#1 Cytokines and HIV and (community acquired pneumonia or pneumonia)

**Database:** Clinical Trials.gov

**Link**:https://clinicaltrials.gov/ct2/results?term=Cytokines+AND+HIV+AND+%28community+acquired+pneumonia+OR+pneumonia%29&type=Obsr&rslt=&recr=&age_v=&age=1&gndr=&hlth=Y&cond=&intr=&titles=&outc=&spons=&lead=&id=&state1=&cntry1=&state2=&cntry2=&state3=&cntry3=&locn=&rcv_s=&rcv_e=&lup_s=&lup_e=

**Search Details:** Cytokines AND HIV AND (community acquired pneumonia OR pneumonia) | Observational Studies | Adult | Studies that accept healthy volunteers

**Database:** Google Scholar

**Link**:https://scholar.google.ca/scholar?start=10&q=Human+adult+pneumonia+cytokine+HIV+pneumonia+%22observational+study%22+OR+%22comparative+study%22+OR+%22clinical+study%22+OR+%22clinical+trial%22+%2218+years+old%22+-%22Cystic+fibrosis%22+-COPD+-cancer+-transplant+-neonate+-child&hl=en&as_sdt=0,5

**Search Details:** Human adult pneumonia cytokine HIV pneumonia "observational study" OR "comparative study" OR "clinical study" OR "clinical trial" "18 years old" -"Cystic fibrosis" -COPD -cancer -transplant -neonate -child

**18. Cytokines AND (lung injury OR lung inflammation OR “lung function” OR “lung function decline”) AND HIV AND (community acquired pneumonia OR pneumonia)**

**Date**: June 1^st^, 2018

**Database:** Pubmed

**Link**: https://www.ncbi.nlm.nih.gov/pubmed/?cmd=HistorySearch&querykey=41

**Search Details:** "cytokines"[MeSH Major Topic] AND (("lung injury"[MeSH Terms] OR ("lung"[All Fields] AND "injury"[All Fields]) OR "lung injury"[All Fields]) OR ("pneumonia"[MeSH Terms] OR

"pneumonia"[All Fields] OR ("lung"[All Fields] AND "inflammation"[All Fields]) OR "lung inflammation"[All Fields]) OR "lung function"[All Fields] OR "lung function decline"[All Fields]) AND ("hiv"[MeSH Terms] OR "hiv"[All Fields]) AND ((("residence characteristics"[MeSH Terms] OR ("residence"[All Fields] AND "characteristics"[All Fields]) OR "residence characteristics"[All Fields] OR "community"[All Fields]) AND acquired[All Fields] AND ("pneumonia"[MeSH Terms] OR "pneumonia"[All Fields])) OR ("pneumonia"[MeSH Terms] OR "pneumonia"[All Fields])) AND ((Clinical Study[ptyp] OR Clinical Trial[ptyp] OR Comparative Study[ptyp] OR Observational Study[ptyp]) AND "humans"[MeSH Terms] AND "adult"[MeSH Terms])

**Database:** CENTRAL

**Link**: http://onlinelibrary.wiley.com/cochranelibrary/search

**Search Details:** Cytokines AND (lung injury OR lung inflammation OR lung function OR lung function decline) AND HIV AND (community acquired pneumonia OR pneumonia)

Description:

ID Search

#1 Cytokines and (lung injury or lung inflammation or lung function or lung function decline) and HIV and (community acquired pneumonia or pneumonia)

**Database:** Clinical Trials.gov

**Link**:https://clinicaltrials.gov/ct2/results?term=Cytokines+AND+%28lung+injury+OR+lung+inflammation+OR+lung+function+OR+lung+function+decline%29+AND+HIV+AND+%28community+acquired+pneumonia+OR+pneumonia%29&type=Obsr&rslt=&recr=&age_v=&age=1&gndr=&hlth=Y&cond=&intr=&titles=&outc=&spons=&lead=&id=&state1=&cntry1=&state2=&cntry2=&state3=&cntry3=&locn=&rcv_s=&rcv_e=&lup_s=&lup_e=

**Search Details:** Cytokines AND (lung injury OR lung inflammation OR lung function OR lung function decline) AND HIV AND (community acquired pneumonia OR pneumonia) | Observational Studies | Adult | Studies that accept healthy volunteers

**Database:** Google Scholar

**Link**:https://scholar.google.ca/scholar?as_q=Human+adult+pneumonia+cytokine+%2218+years+old%22+HIV+pneumonia&as_epq=lung+injury&as_oq=%22observational+study%22+%22comparative+study%22+%22clinical+study%22+%22clinical+trial%22&as_eq=%22Cystic+fibrosis%22+COPD+cancer+transplant+neonate+child&as_occt=any&as_sauthors=&as_publication=&as_ylo=&as_yhi=&btnG=&hl=en&as_sdt=0%2C5

**Search Details:** Human adult pneumonia cytokine "18 years old" HIV pneumonia "observational study" OR "comparative study" OR "clinical study" OR "clinical trial" "lung injury" -"Cystic fibrosis" -COPD -cancer -transplant -neonate -child

**19. Cytokines AND (lung injury OR lung inflammation OR “lung function” OR “lung function decline”) AND (HIV OR community acquired pneumonia OR pneumonia)**

**Date**: June 1^st^, 2018

**Database:** Pubmed

**Link**: https://www.ncbi.nlm.nih.gov/pubmed/?cmd=HistorySearch&querykey=40

**Search Details:** "cytokines"[MeSH Major Topic] AND (("lung injury"[MeSH Terms] OR ("lung"[All Fields] AND "injury"[All Fields]) OR "lung injury"[All Fields]) OR ("pneumonia"[MeSH Terms] OR "pneumonia"[All Fields] OR ("lung"[All Fields] AND "inflammation"[All Fields]) OR "lung inflammation"[All Fields]) OR "lung function"[All Fields] OR "lung function decline"[All Fields]) AND (("hiv"[MeSH Terms] OR "hiv"[All Fields]) OR (("residence characteristics"[MeSH Terms] OR ("residence"[All Fields] AND "characteristics"[All Fields]) OR "residence characteristics"[All Fields] OR "community"[All Fields]) AND acquired[All Fields] AND ("pneumonia"[MeSH Terms] OR "pneumonia"[All Fields])) OR ("pneumonia"[MeSH Terms] OR "pneumonia"[All Fields])) AND ((Clinical Study[ptyp] OR Clinical Trial[ptyp] OR Comparative Study[ptyp] OR Observational Study[ptyp]) AND "humans"[MeSH Terms] AND "adult"[MeSH Terms])

**Database:** CENTRAL

**Link**: http://onlinelibrary.wiley.com/cochranelibrary/search

**Search Details:** Cytokines and (lung injury or lung inflammation or "lung function" or "lung function decline") and (HIV or community acquired pneumonia or pneumonia)

Description:

ID Search

#1 Cytokines and (lung injury or lung inflammation or "lung function" or "lung function decline") and (HIV or community acquired pneumonia or pneumonia)

**Database:** Clinical Trials.gov

**Link**:https://clinicaltrials.gov/ct2/results?term=Cytokines+AND+%28lung+injury+OR+lung+inflammation+OR+“lung+function”+OR+“lung+function+decline”%29+AND+%28HIV+OR+community+acquired+pneumonia+OR+pneumonia%29&type=Obsr&rslt=&recr=&age_v=&age=1&gndr=&hlth=Y&cond=&intr=&titles=&outc=&spons=&lead=&id=&state1=&cntry1=&state2=&cntry2=&state3=&cntry3=&locn=&rcv_s=&rcv_e=&lup_s=&lup_e=

**Search Details:** Cytokines AND (lung injury OR lung inflammation OR “lung function” OR “lung function decline”) AND (HIV OR community acquired pneumonia OR pneumonia) | Observational Studies | Adult | Studies that accept healthy volunteers

**Database:** Google Scholar

**Link**:https://scholar.google.ca/scholar?as_q=Human+adult+pneumonia+cytokine&as_epq=%22lung+injury%22+%2218+years+old%22&as_oq=HIV+pneumonia+%22observational+study%22+%22comparative+study%22+%22clinical+study%22+%22clinical+trial%22&as_eq=%22Cystic+fibrosis%22+COPD+cancer+transplant+neonate+child&as_occt=any&as_sauthors=&as_publication=&as_ylo=&as_yhi=&btnG=&hl=en&as_sdt=0%2C5

**Search Details:** Human adult pneumonia cytokine HIV OR pneumonia OR "observational study" OR "comparative study" OR "clinical study" OR "clinical trial" "lung injury" "18 years old" -"Cystic fibrosis" -COPD -cancer -transplant -neonate -child

**20. Cytokines AND (community-acquired pneumonia OR pneumonia)**

**Date**: June 1^st^, 2018

**Database:** Pubmed

**Link**: https://www.ncbi.nlm.nih.gov/pubmed

**Search Details:** "cytokines"[MeSH Major Topic] AND ((community-acquired[All Fields] AND ("pneumonia"[MeSH Terms] OR "pneumonia"[All Fields])) OR ("pneumonia"[MeSH Terms] OR "pneumonia"[All Fields])) AND ((Clinical Study[ptyp] OR Clinical Trial[ptyp] OR Comparative Study[ptyp] OR Observational Study[ptyp]) AND "humans"[MeSH Terms] AND "adult"[MeSH Terms])

**Database:** CENTRAL

**Link**: http://onlinelibrary.wiley.com/cochranelibrary/search

**Search Details:** Cytokines AND (community-acquired pneumonia OR pneumonia)

Description:

ID Search

#1 Cytokines and (community-acquired pneumonia or pneumonia)

**Database:** Clinical Trials.gov

**Link**: https://clinicaltrials.gov/ct2/results?term=Cytokines+AND+%28community-acquired+pneumonia+OR+pneumonia%29&type=Obsr&rslt=&recr=&age_v=&age=1&gndr=&hlth=Y&cond=&intr=&titles=&outc=&spons=&lead=&id=&state1=&cntry1=&state2=&cntry2=&state3=&cntry3=&locn=&rcv_s=&rcv_e=&lup_s=&lup_e=

**Search Details:** Cytokines AND (community-acquired pneumonia OR pneumonia) | Observational Studies | Adult | Studies that accept healthy volunteers

**Database:** Google Scholar

**Link**:https://scholar.google.ca/scholar?as_q=Human+adult+pneumonia+cytokine&as_epq=18+years+old+&as_oq=%22observational+study%22+%22comparative+study%22+%22clinical+study%22+%22clinical+trial%22&as_eq=%22Cystic+fibrosis%22+COPD+cancer+transplant+neonate+child&as_occt=any&as_sauthors=&as_publication=&as_ylo=&as_yhi=&btnG=&hl=en&as_sdt=0%2C5

**Search Details:** Human adult pneumonia cytokine "observational study" OR "comparative study" OR "clinical study" OR "clinical trial" "18 years old" -"Cystic fibrosis" -COPD -cancer -transplant -neonate -child

**21. Biomarkers AND (lung injury OR lung inflammation OR “lung function” OR “lung function decline”) AND (HIV OR community acquired pneumonia OR pneumonia)**

**Date**: June 1^st^, 2018

**Database:** Pubmed

**Link**: https://www.ncbi.nlm.nih.gov/pubmed/?cmd=HistorySearch&querykey=38

**Search Details:** "biomarkers"[MeSH Major Topic] AND (("lung injury"[MeSH Terms] OR ("lung"[All Fields] AND "injury"[All Fields]) OR "lung injury"[All Fields]) OR ("pneumonia"[MeSH Terms] OR "pneumonia"[All Fields] OR ("lung"[All Fields] AND "inflammation"[All Fields]) OR "lung inflammation"[All Fields]) OR "lung function"[All Fields] OR "lung function decline"[All Fields]) AND (("hiv"[MeSH Terms] OR "hiv"[All Fields]) OR (("residence characteristics"[MeSH Terms] OR ("residence"[All Fields] AND "characteristics"[All Fields]) OR "residence characteristics"[All Fields] OR "community"[All Fields]) AND acquired[All Fields] AND ("pneumonia"[MeSH Terms] OR "pneumonia"[All Fields])) OR ("pneumonia"[MeSH Terms] OR

"pneumonia"[All Fields])) AND ((Clinical Study[ptyp] OR Clinical Trial[ptyp] OR Comparative Study[ptyp] OR Observational Study[ptyp]) AND "humans"[MeSH Terms] AND "adult"[MeSH Terms])

**Database:** CENTRAL

**Link**: http://onlinelibrary.wiley.com/cochranelibrary/search

**Search Details:** Biomarkers AND (lung injury OR lung inflammation OR "lung function" OR "lung function decline") AND (HIV OR community acquired pneumonia OR pneumonia)

Description:

ID Search

#1 Biomarkers and (lung injury or lung inflammation or "lung function" or "lung function decline") and (HIV or community acquired pneumonia or pneumonia)

**Database:** Clinical Trials.gov

**Link**:https://clinicaltrials.gov/ct2/results?term=Biomarkers+AND+%28lung+injury+OR+lung+inflammation+OR+“lung+function”+OR+“lung+function+decline”%29+AND+%28HIV+OR+community+acquired+pneumonia+OR+pneumonia%29&type=Obsr&rslt=&recr=&age_v=&age=1&gndr=&hlth=Y&cond=&intr=&titles=&outc=&spons=&lead=&id=&state1=&cntry1=&state2=&cntry2=&state3=&cntry3=&locn=&rcv_s=&rcv_e=&lup_s=&lup_e=

**Search Details:** Biomarkers AND (lung injury OR lung inflammation OR “lung function” OR “lung function decline”) AND (HIV OR community acquired pneumonia OR pneumonia) | Observational Studies | Adult | Studies that accept healthy volunteers

**Database:** Google Scholar

**Link**:https://scholar.google.ca/scholar?as_q=human+adult+18+years+old+biomarker+%22lung+injury%22&as_epq=lung+function&as_oq=%22observational+study%22+%22comparative+study%22+%22clinical+study%22+%22clinical+trial%22+HIV+pneumonia&as_eq=%22Cystic+fibrosis%22+COPD+cancer+transplant+neonate+child&as_occt=any&as_sauthors=&as_publication=&as_ylo=&as_yhi=&btnG=&hl=en&as_sdt=0%2C5

**Search Details:** human adult 18 years old biomarker "lung injury" "observational study" OR "comparative study" OR "clinical study" OR "clinical trial" OR HIV OR pneumonia "lung function" -"Cystic fibrosis" -COPD -cancer -transplant -neonate -child

**22. Biomarkers AND (lung injury OR lung inflammation OR “lung function” OR “lung function decline”) AND HIV AND (community acquired pneumonia OR pneumonia)**

**Date**: June 1^st^, 2018

**Database:** Pubmed

**Link**: https://www.ncbi.nlm.nih.gov/pubmed/?cmd=HistorySearch&querykey=37

**Search Details:** "biomarkers"[MeSH Major Topic] AND (("lung injury"[MeSH Terms] OR ("lung"[All Fields] AND "injury"[All Fields]) OR "lung injury"[All Fields]) OR ("pneumonia"[MeSH Terms] OR "pneumonia"[All Fields] OR ("lung"[All Fields] AND "inflammation"[All Fields]) OR "lung inflammation"[All Fields]) OR "lung function"[All Fields] OR "lung function decline"[All Fields]) AND ("hiv"[MeSH Terms] OR "hiv"[All Fields]) AND ((("residence characteristics"[MeSH Terms] OR ("residence"[All Fields] AND "characteristics"[All Fields]) OR "residence

characteristics"[All Fields] OR "community"[All Fields]) AND acquired[All Fields] AND ("pneumonia"[MeSH Terms] OR "pneumonia"[All Fields])) OR ("pneumonia"[MeSH Terms] OR "pneumonia"[All Fields])) AND ((Clinical Study[ptyp] OR Clinical Trial[ptyp] OR Comparative Study[ptyp] OR Observational Study[ptyp]) AND "humans"[MeSH Terms] AND "adult"[MeSH Terms])

**Database:** CENTRAL

**Link**: http://onlinelibrary.wiley.com/cochranelibrary/search

**Search Details:** Biomarkers and (lung injury or lung inflammation or "lung function" or "lung function decline") and HIV and (community acquired pneumonia or pneumonia)

Description:

ID Search

#1 Biomarkers and (lung injury or lung inflammation or "lung function" or "lung function decline") and HIV and (community acquired pneumonia or pneumonia)

**Database:** Clinical Trials.gov

**Link**:https://clinicaltrials.gov/ct2/results?term=Biomarkers+AND+%28lung+injury+OR+lung+inflammation+OR+“lung+function”+OR+“lung+function+decline”%29+AND+HIV+AND+%28community+acquired+pneumonia+OR+pneumonia%29&type=Obsr&rslt=&recr=&age_v=&age=1&gndr=&hlth=Y&cond=&intr=&titles=&outc=&spons=&lead=&id=&state1=&cntry1=&state2=&cntry2=&state3=&cntry3=&locn=&rcv_s=&rcv_e=&lup_s=&lup_e=

**Search Details:** Biomarkers AND (lung injury OR lung inflammation OR “lung function” OR “lung function decline”) AND HIV AND (community acquired pneumonia OR pneumonia) | Observational Studies | Adult | Studies that accept healthy volunteers

**Database:** Google Scholar

**Link**:https://scholar.google.ca/scholar?as_q=human+adult+18+years+old+biomarker+HIV+pneumonia+%22lung+function%22&as_epq=lung+injury&as_oq=%22observational+study%22+%22comparative+study%22+%22clinical+study%22+%22clinical+trial%22&as_eq=%22Cystic+fibrosis%22+COPD+cancer+transplant+neonate+child&as_occt=any&as_sauthors=&as_publication=&as_ylo=&as_yhi=&btnG=&hl=en&as_sdt=0%2C5

**Search Details:** Human adult 18 years old biomarker HIV pneumonia "lung function" "observational study" OR "comparative study" OR "clinical study" OR "clinical trial" "lung injury" --"Cystic fibrosis" -COPD -cancer -transplant -neonate -child

**23. Biomarkers AND HIV AND (community acquired pneumonia OR pneumonia)**

**Date**: June 1^st^, 2018

**Database:** Pubmed

**Link**:https://www.ncbi.nlm.nih.gov/pubmed/?term=Biomarkers+%5BMAJR%5D+AND+HIV+AND+(community+acquired+pneumonia+OR+pneumonia)

**Search Details:** "biomarkers"[MeSH Major Topic] AND ("hiv"[MeSH Terms] OR "hiv"[All Fields]) AND ((("residence characteristics"[MeSH Terms] OR ("residence"[All Fields] AND "characteristics"[All Fields]) OR "residence characteristics"[All Fields] OR "community"[All Fields]) AND acquired[All Fields] AND ("pneumonia"[MeSH Terms] OR "pneumonia"[All Fields]))

OR ("pneumonia"[MeSH Terms] OR "pneumonia"[All Fields])) AND ((Clinical Study[ptyp] OR Clinical Trial[ptyp] OR Comparative Study[ptyp] OR Observational Study[ptyp]) AND "humans"[MeSH Terms] AND "adult"[MeSH Terms])

**Database:** CENTRAL

**Link**: http://onlinelibrary.wiley.com/cochranelibrary/search

**Search Details:** Biomarkers and HIV and (community acquired pneumonia or pneumonia)

Description:

ID Search

#1 Biomarker and HIV and (community acquired pneumonia or pneumonia)

**Database:** Clinical Trials.gov

**Link**:https://clinicaltrials.gov/ct2/results?term=Biomarkers+AND+HIV+AND+%28community+acquired+pneumonia+OR+pneumonia%29&type=Obsr&rslt=&recr=&age_v=&age=1&gndr=&hlth=Y&cond=&intr=&titles=&outc=&spons=&lead=&id=&state1=&cntry1=&state2=&cntry2=&state3=&cntry3=&locn=&rcv_s=&rcv_e=&lup_s=&lup_e=

**Search Details:** Biomarkers AND HIV AND (community acquired pneumonia OR pneumonia) | Observational Studies | Adult | Studies that accept healthy volunteers

**Database:** Google Scholar

**Link**: https://scholar-google-com.uml.idm.oclc.org/scholar?as_q=human+adult+18+years+old+biomarker+HIV+pneumonia&as_epq=&as_oq=%22observational+study%22+%22comparative+study%22+%22clinical+study%22+%22clinical+trial%22&as_eq=%22Cystic+fibrosis%22+COPD+cancer+transplant+neonate+child&as_occt=any&as_sauthors=&as_publication=&as_ylo=2014&as_yhi=2018&btnG=&hl=en&as_sdt=1%2C36

**Search Details:** Human adult 18 years old biomarker HIV pneumonia "observational study" OR "comparative study" OR "clinical study" OR "clinical trial" -"Cystic fibrosis" -COPD -cancer -transplant -neonate -child

**24. Biomarkers AND (community-acquired pneumonia OR pneumonia)**

**Date**: June 1^st^, 2018

**Database:** Pubmed

**Link**: https://www.ncbi.nlm.nih.gov/pubmed

**Search Details:** "biomarkers"[MeSH Major Topic] AND ((community-acquired[All Fields] AND ("pneumonia"[MeSH Terms] OR "pneumonia"[All Fields])) OR ("pneumonia"[MeSH Terms] OR "pneumonia"[All Fields])) AND ((Clinical Study[ptyp] OR Clinical Trial[ptyp] OR Comparative Study[ptyp] OR Observational Study[ptyp]) AND "humans"[MeSH Terms] AND "adult"[MeSH Terms])

**Database:** CENTRAL

**Link**:http://onlinelibrary.wiley.com/cochranelibrary/search/

**Search Details:** Biomarkers AND (community-acquired pneumonia OR pneumonia)

Description:

ID Search

#1 Biomarkers and (community-acquired pneumonia or pneumonia)

**Database:** Clinical Trials.gov

**Link**: https://clinicaltrials.gov/ct2/results?term=Biomarkers+AND+%28community-acquired+pneumonia+OR+pneumonia%29&type=Obsr&rslt=&recr=&age_v=&age=1&gndr=&hlth=Y&cond=&intr=&titles=&outc=&spons=&lead=&id=&state1=&cntry1=&state2=&cntry2=&state3=&cntry3=&locn=&rcv_s=&rcv_e=&lup_s=&lup_e=

**Search Details:** Biomarkers AND (community-acquired pneumonia OR pneumonia) | Observational Studies | Adult | Studies that accept healthy volunteers

**Database:** Google Scholar

**Link**: https://scholar.google.ca/scholar?as_q=Human+adult++biomarker+pneumonia&as_epq=18+years+old&as_oq=%22observational+study%22+%22comparative+study%22+%22clinical+study%22+%22clinical+trial%22&as_eq=%22Cystic+fibrosis%22+COPD+cancer+transplant+neonate+child&as_occt=any&as_sauthors=&as_publication=&as_ylo=&as_yhi=&btnG=&hl=en&as_sdt=0%2C5

**Search Details:** Human adult biomarker pneumonia "observational study" OR "comparative study" OR "clinical study" OR "clinical trial" "18 years old" -"Cystic fibrosis" -COPD -cancer -transplant -neonate -child
